# An improved cytological assay for R-loop detection in *Saccharomyces cerevisiae* utilizing a catalytically inactive RNase H

**DOI:** 10.1093/g3journal/jkaf072

**Published:** 2025-04-10

**Authors:** Jordan Sanders, Zainab Hakeem, Anthony Schwacha

**Affiliations:** Department of Biological Sciences, University of Pittsburgh, Pittsburgh, PA 15260, USA; Department of Biochemistry and Molecular Biology, University of Georgia, Athens, GA 30602, USA; Department of Biological Sciences, University of Pittsburgh, Pittsburgh, PA 15260, USA

**Keywords:** dRnh1-GFP, R-loop, RNA/DNA hybrid, RNase H1, transcription–replication conflict, yeast

## Abstract

R-loops (RNA/DNA hybrids) are caused by defects in RNA transcription or processing, and their level heavily correlates with genome instability and human disease. Most current yeast methods for R-loop analysis use fixed or disrupted cells probed with an R-loop-specific antibody (S9.6), and relatively few cytological methods are available to visualize R-loops in living cells. Here, we present a simplified cytological method for R-loop detection in live cells of the yeast *Saccharomyces cerevisiae* using a catalytically inactive RNase H1 protein coupled to GFP (dRnh1-GFP reporter). In cells lacking the endogenous RNase H1 gene, reporter expression generates bright nuclear foci that colocalize with R-loops as defined by S9.6 immunocytology. We find that our dRnh1-GFP reporter system can sensitively identify and track changes in R-loop levels induced by various mutations and small molecules known to increase R-loops. Given its ease of use and superior R-loop specificity relative to S9.6, the dRnh1-GFP reporter is suitable for use in high-throughput experiments and presents an exciting opportunity to deepen our understanding of R-loops and their regulatory mechanisms.

## Introduction

R-loops are 3-stranded structures in which a single-stranded RNA anneals to an extensive tract (∼100–2,700 bp) (Malig *et al*. 2020) of double-stranded DNA, displacing the complimentary DNA strand and generating a “loop” structure (Costantino and Koshland 2015). The displacement of the complimentary DNA strand leaves it in a vulnerable single-stranded state, increasing the risk of DNA breaks. Furthermore, as they can cause topological impediments to critical processes such as DNA replication (Costantino and Koshland 2015), R-loops may additionally cause double-strand breaks (chromosome fragility) by impeding fork progression at highly transcribed genes (Costantino and Koshland 2015; Vijayraghavan *et al*. 2016) and in regions of DNA that are inherently difficult for replication machinery to traverse (Costantino and Koshland 2018; Brambati *et al*. 2020). Despite their roles in genome instability, in some circumstances R-loops have been proposed to positively modulate genomic integrity and transcription. So-called “physiological” R-loops preserve telomere integrity (Costantino and Koshland 2015; Rivosecchi *et al*. 2024), stimulate DNA recombination repair (Costantino and Koshland 2015; Teng *et al*. 2018), and promote mitochondrial DNA replication (Lee and Clayton 1998). They also regulate transcription at initiation and termination sites (Chen *et al*. 2015; Yang *et al*. 2019), as well as prevent DNA methylation at CpG islands (Ginno *et al*. 2012; Costantino and Koshland 2015).

Abnormal R-loop accumulation can be caused by a variety of cellular malfunctions. Defects in transcription or RNA export from the nucleus increase R-loops in both yeast and humans (Brambati *et al*. 2020) and are associated with neurological disorders and autoimmune disease (Richard and Manley 2017; Bradley and Savage 2023). In addition, malfunctions in the replication fork (Vijayraghavan *et al*. 2016) and associated topoisomerases (Patel *et al*. 2022) also increase R-loop levels and concomitantly increase double-stranded DNA breaks in transcription–replication conflict zones (Chang and Stirling 2017). Finally, defects in various enzymes dedicated to the removal of R-loops such as DNA repair helicases (Brambati *et al*. 2020), and RNA/DNA nucleases such as RNase H1 (Amon and Koshland 2016) increase R-loop levels and coordinately cause diseases associated with genomic instability (Richard and Manley 2017).

The wide roles of R-loops in both DNA damage and repair necessitate their study. Unfortunately, currently only 2 tools are commonly used to detect and quantify R-loops. The first is a monoclonal antibody that binds RNA/DNA hybrids (S9.6) and is used for immunocytological R-loop detection (Wahba *et al*. 2011), chromatin immunoprecipitation-based genomic analysis (Wahba *et al*. 2016; Sanz and Chedin 2019), and dot blot quantification (Ramirez *et al*. 2021). However, S9.6 also has a relatively high affinity for dsRNA (Crossley *et al*. 2021; Smolka *et al*. 2021) and is unsuitable for R-loop detection in living cells. A second approach, initially engineered by Nguyen *et al*. (2017), utilizes a catalytically “dead” RNase H1-GFP reporter (dRnh1-GFP) that binds to R-loops without degrading them. This construct, and other iterations of it (Chen *et al*. 2017; Martin *et al*. 2023), is advantageous for its ability to detect R-loops in living cells (Nguyen *et al*. 2017; Shen *et al*. 2017). The dRnh1-GFP protein can also be purified and used much like an antibody for imaging in fixed cells (Crossley *et al*. 2021). Substitution of a V5 tag in place of GFP allows for its use in chromatin immunoprecipitation-based approaches for genomic R-loop mapping (Chen *et al*. 2017). However, the success of the dRnh1-GFP system has yet to be extended to other experimental organisms where their unique advantages can be leveraged.

*RNH1* is an R-loop-specific nuclease with evolutionarily conserved function and protein sequence. RNA/DNA hybrids are recognized by Rnh1's N-terminus hybrid-binding domain (Supplementary Fig. 1) (Cerritelli and Crouch 2009), while the C-terminus catalytic domain encodes RNase H activity and conserved active site acidic residues (Hyjek *et al*. 2019). These 2 domains are connected by a flexible linker that allows the hybrid-binding domain to remain stationarily bound to nucleic acid while leaving the nuclease domain free to cleave RNA at multiple sites (Cerritelli and Crouch 2009). Yeast Rnh1 differs from its human homolog in that it lacks a mitochondrial targeting sequence (Cerritelli and Crouch 2009), a feature that ultimately is beneficial for cytology since there is no possibility of interfering mitochondrial signal when Rnh1 is tagged with a GFP reporter.

Here, we report on the engineering and efficacy of a dRnh1-GFP system in *Saccharomyces cerevisiae.* This integrated and inducible dRnh1-GFP reporter can detect changes in R-loop levels in living cells induced by both mutation and drug treatment. The simplicity of this system allows for analysis of R-loops at a population level in real time. The myriad of genetic tools available in yeast make this dRnh1-GFP system uniquely suitable for high throughput genetic and drug screens, providing great potential for future R-loop research.

## Materials and methods

### Yeast, growth, and reagents

All *S. cerevisiae* strains are isogenic derivatives of SEY6210, carry deletions to key multidrug transporters (*YOR1*, *SNQ2*, and *PDR5*) that facilitate small molecule sensitivity (Kolaczkowski *et al*. 1998) and contain the β-estradiol (BED)–responsive Act1-GEV transcriptional induction system (McIsaac *et al*. 2011). Strains and plasmids were constructed using standard budding yeast methodology (Supplementary Table 1 and 2). Oligos used for strain construction are listed in Supplementary Table 3, and construction details are available upon request. The VHL-mCherry and *BTN2* deletion constructs were kind gifts from Bernd Bukau (University of Heidelberg). Sources and concentrations of antibodies, small molecule inhibitors, and other reagents used in this study are listed in Supplementary Table 4. For growth rate experiments, log-phase cultures were diluted to OD_600_ = 0.05 and seeded in Falcon clear-bottomed 96-well plates (353072) containing informative concentrations of BED. Plates were incubated at 30°C for 24 h with continuous agitation and hourly recording of OD_600_ using a BioTek Cytation 5 multimode plate reader.

### Construction of the dRnh1-GFP reporter

The dRnh1-GFP construct was assembled using Gateway cloning (Alberti *et al*. 2007). In brief, *attB1* and *attB2* sites were introduced immediately upstream and downstream of wild-type *RNH1* via PCR amplification, which facilitated their eventual integration into the destination vector pAG306GAL-ccdB-EGFP (Addgene 14187) (Alberti *et al*. 2007) between an inducible Gal promoter and an in-frame C-terminal EGFP epitope tag. Site-directed mutagenesis was then performed to either inactivate Rnh1's catalytic domain (D264N) or to inactivate the hybrid-binding domain (W22A, K38A, and K39A). The resulting vectors were linearized with the restriction nuclease NcoI and integrated into budding yeast at the *URA3* locus.

### Western blot analysis

Western blots were conducted using standard methods. The dRnh1-GFP reporter was detected using Roche mouse monoclonal α-GFP (11814460001). Glucose 6-phosphate dehydrogenase (G6PDH) served as a loading control and was detected using Sigma A9521 polyclonal rabbit α-G6PDH. Chemiluminescence was conducted with SuperSignal West Pico PLUS Chemiluminescent Substrate spiked with 10% ProSignal Dura Low-Femtogram ECL Reagent, images were developed on an Amersham Imager 600, and results were quantified using FIJI software (Schindelin *et al*. 2012).

### dRnh1-GFP live-cell focus assay

Indicated strains were inoculated from single colonies and grown overnight with agitation at 30°C in YPD (yeast peptone dextrose) + 100 μg/mL Nourseothricin (Goldbio, Cat. # N-500-1) to select for the act1-GEV construct. Log-phase strains were then diluted to OD_600 nM_ = 0.05, treated with the desired concentration of BED (Sigma E8875), and then incubated for 4 h with agitation in YPD + BED at 30°C prior to harvest. One milliliter of the sample was sonicated, centrifuged, and resuspended in 40% glycerol. One microgram per milliliter of DAPI was included with the glycerol if representative images were being taken of the cells. Cells were immediately analyzed using fluorescence microscopy with a Zeiss Axioskop 40 and Zeiss filter sets 2 (blue), 9 (green), or 20 (red). A Zeiss Axio Scope A1 microscope with Zeiss filter sets 49 (blue), 38, (green), and 43 (red) was used to visualize cells expressing VHL-mCherry. A minimum of 100 cells were counted in each replicate to measure the population average of cells displaying at least one dRnh1-GFP focus. A cell was considered to contain a dRnh1-GFP focus if it contained a roughly circular and clearly discreet region of elevated fluorescence compared with the surrounding nucleus. Unless otherwise noted in the relevant figure legend, all assays were independently repeated ≥3 times, and the averaged value and standard error of the mean were reported. Statistical differences between the control and experimental samples were tested by 2-way ANOVA using GraphPad PRISM software version 10.3.0 (Dunnett's multiple comparison's test, adjusted *P*-values were reported). To determine focus characteristics and nucleus dRnh1-GFP fluorescence, pixel intensity was plotted across a drawn line intersecting the focus and surrounding nucleus. Focus diameter and area was calculated with FIJI software (Schindelin *et al*. 2012) using the peak width at 33% of its total pixel intensity.

### Yeast chromatin spreads and colocalization assays

A detailed protocol for the harvesting and labeling of chromatin spreads with S9.6 α-RNA/DNA hybrid antibody and the analysis methods for establishing colocalization between S9.6 and dRnh1-GFP can be found in Supplementary Methods. In brief, log-phase cells were diluted to OD_600 nM_ = 0.05, induced with 2 nM BED for 4 h, and were harvested for immunofluorescence (IF) following a previously developed protocol (Wahba *et al*. 2011). The resulting chromatin spreads were probed with S9.6 (Phillips *et al*. 2013) and imaged using a Zeiss Axioskop 40 with Zeiss AxioCam HRM. Image exposure time and brightness/contrast settings were held constant for all images within an experimental set.

Captured images in an experimental set were processed in parallel with FIJI image analysis software (Schindelin *et al*. 2012). Low-level background in all channels was subtracted using default settings (rolling ball radius 50 pixels). Pearson's colocalization coefficient was derived from these modified images using the JACoP plugin (Bolte and Cordelieres 2006). For visual scoring of cells, images were further thresholded to reduce background fluorescence from being scored as visible signal. Nonspecific signal from secondary antibody was subtracted from all images by using no-primary control images to set threshold values. Auto-fluorescence in the green channel was similarly removed based on control cells grown in the absence of BED. Cells were deemed to have overlapping dRnh1-GFP/S9.6 signal only if the 2 signals overlap the same space *and* possess a similar size/pattern.

## Results

### dRnh1-GFP reporter construction

The binding of multiple copies of a GFP-tagged reporter to a nuclear target should produce visibly discreet foci. In budding yeast, GFP-tagged DNA recombination/repair factors form foci and have been used as reporters [e.g. the use of Rad52-YFP to study DNA double-strand break repair (Lisby *et al*. 2003)]. We endeavored to engineer an analogous reporter to detect R-loop accumulation using *S. cerevisiae*  Rnh1.

Our proposed reporter system (dRnh1-GFP) needed to address several experimental issues: (1) Neither the human nor yeast wild-type Rnh1 form discrete nuclear foci (Huh *et al*. 2003; Shen *et al*. 2017) presumably because R-loop turnover is too rapid. We therefore sought a suitable mutation in the nuclease domain to block R-loop repair but not binding. Related human cell systems solved this problem by mutating a conserved aspartate in the *RNH1* nuclease domain to an asparagine (D210N) (Nguyen *et al*. 2017); in *S. cerevisiae* the homologous aspartate corresponds to D264 (Supplementary Fig. 1). (2) To limit any possible deleterious effects of a catalytically defective rnh1 protein, our reporter system would utilize an inducible promoter to allow fine-tuning of its expression (McIsaac *et al*. 2011).

The starting RNH1-GFP reporter plasmid contained wild-type *RNH1* expressed from a galactose inducible promoter and C-terminally tagged with enhanced GFP (EGFP) (*Materials and Methods*). As shown below, this reporter compliments a *rnh1Δ* mutant, indicating that the GFP tag does not interfere with gene function. The wild-type reporter was then mutagenized to incorporate the D264N mutation and create the catalytically defective reporter (dRnh1-GFP). As the expression of galactose-inducible genes in *S. cerevisiae* is difficult to fine-tune and is inhibited by the presence of glucose in the growth media (Adams 1972), we expressed our reporter using a previously developed artificial induction system (Act1-GEV) that facilitates the expression of galactose-inducible genes using the synthetic inducer BED (McIsaac *et al*. 2011). Both the dRnh1-GFP reporter and Act1-GEV were then stably integrated into the genomes of a series of *S. cerevisiae* strains (discussed below). Reporter expression was assayed via Western blot. In the absence of BED, little dRnh1-GFP was detected (Supplementary Fig. 2a). However, upon BED induction, robust and tunable expression of dRnh1-GFP was observed (Supplementary Fig. 2a and b).

### dRnh1-GFP forms discreet nuclear foci only in the absence of endogenous *RNH1*

**Fig. 1.**
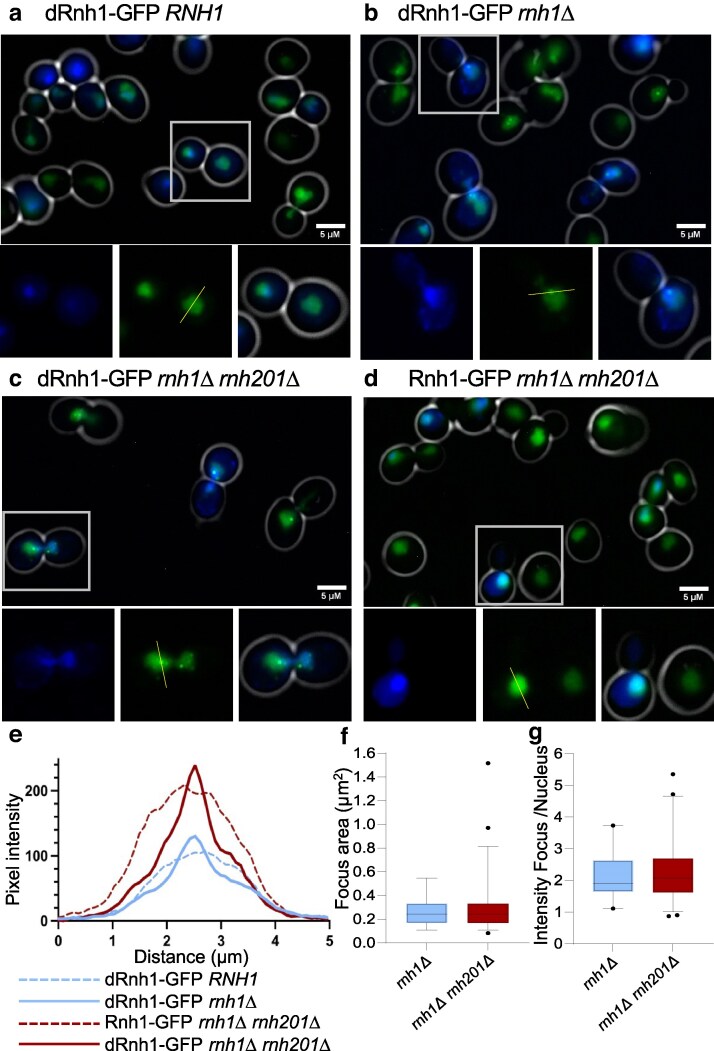
The dRnh1-GFP reporter forms nuclear foci. a–d) Representative images of live cells treated with 2 nM BED for 4 h. DAPI-stained DNA is shown in blue, Rhn1-EGFP reporters in green, and bright field–derived cell walls in white. Histograms in e) correspond to the relative pixel intensity of GFP signal along the path of the yellow lines present in each inset image. a) Wild-type strain expressing dRnh1-GFP. b) *rnh1Δ* strain expressing dRnh1-GFP. c) *rnh1Δ rnh201Δ* strain expressing dRnh1-GFP. d) *rnh1Δ rnh201Δ* strain expressing a wild-type Rnh1-GFP. e) Histograms of pixel intensity across the drawn yellow lines depicted in the insets of a–d). f) Box plot of the area of dRnh1-GFP foci in both *rnh1Δ* and *rnh1Δ rnh201Δ* strains. Box and bars represent 2.5–97.5th percentile. g) Box plot of pixel intensity of a given dRnh1-GFP focus divided by pixel intensity of the surrounding nucleus. Box and bars represent 2.5–97.5th percentile.

Cytology of the dRnh1-GFP reporter was analyzed in live cells using fluorescence microscopy. Reporter expression was uniform among the yeast population, as following a 4-h induction with 2 nM BED ≥97% of cells in the population typically exhibit green nuclear fluorescence. Although all tested strains demonstrated such inducible fluorescence (Fig. 1a–d), only strains lacking the wild-type *RNH1* gene demonstrated discreet nuclear foci in a subset of cells (Fig. 1b and c). We note that in the *rnh1Δ* background, nuclei typically contain only a single visible focus, with few nuclei containing multiple foci (Fig. 1b).

Wild-type *RNH1* cells may lack dRnh1-GFP foci simply because R-loop levels are very low in such cells (Wahba *et al*. 2016). To test this possibility, we sought a way to elevate R-loops levels independent of *rnh1Δ*. Budding yeast express an additional and partially redundant multisubunit RNaseH dedicated to R-loop repair, with the catalytic subunit corresponding to *RNH201* (Wahba *et al*. 2011; Wahba *et al*. 2016). Mutations in Rnh201 increase R-loop levels relative to the single *rnh1Δ* mutant (Wahba *et al*. 2016). We deleted this gene to generate the *rnh1Δ rnh201Δ* double deletion strain and assayed it for foci following expression of either the dRnh1-GFP or wild-type reporter (Rnh1-GFP) (Fig. 1c and d). Western blot analysis of dRnh1-GFP expression in *rnh1Δ rnh201Δ* confirmed that the reporter expressed at statistically similar levels as a function of [BED] compared with the *rnh1Δ* strain (Supplementary Fig. 2a and b).

In the double-mutant strain, foci formed efficiently upon expression of the dRnh1-GFP reporter (Fig. 1c). These foci were nearly identical to those observed in the *rnh1Δ* single mutant, although multiple foci/cells are more commonly observed in the double mutant (Fig. 1b and c). In contrast, foci did not form when the wild-type Rnh1-GFP reporter was expressed in the double-mutant strain (Fig. 1d). As the *rnh2*01*Δ* mutation elevates R-loop levels even in the presence of a wild-type *RNH1* gene (Amon and Koshland 2016; Wahba *et al*. 2016), this experiment strongly suggests that the wild-type Rnh1 enzyme prevents focus formation by out-competing the dRnh1-GFP reporter, not because *RNH1* strains inherently lack R-loops (discussed further below). Unless otherwise noted, all subsequent experiments were conducted in strains deleted for the endogenous *RNH1*.

Micrographs of both the *rnh1Δ* and *rnh1Δ rnh201Δ* mutants were analyzed to determine the general properties of dRnh1-GFP foci. Strains were grown under identical conditions, and focus properties were quantified using FIJI software (*Materials and Methods*). To examine fluorescence intensity, lines were drawn through individual foci or through nuclei if foci were absent (Fig. 1a–d insets), and histograms were generated across each line (see Fig. 1e for representative histograms). Cells lacking foci demonstrated a broad fluorescence across the entire nucleus (Fig. 1a, d, and e). For cells with foci, the average focus size (∼0.25 µm^2^) did not differ between the 2 examined mutants (Fig. 1f). The average focus was ∼1.8–2.6 times brighter than the surrounding nuclear fluorescence for both mutants (Fig. 1e and g), although a subset of nuclei in the *rnh1Δ rnh201Δ* double-mutant strain trended toward larger and brighter foci compared with *rnh1Δ* (Fig. 1f and g). In summary, focus characteristics are quite reproducible in terms of signal to background and size.

### Optimizing assay conditions

To develop a reproducible and quantitative assay, we optimized the inducer concentration and induction time. We found that the fraction of cells containing 1 or more dRnh1-GFP foci peaked and leveled off by 4 h post BED induction (Supplementary Fig. 2c and d). We then treated the *rnh1Δ* and *rnh1Δ rnh201Δ* strains with a range of BED concentrations to determine the minimal amount of BED needed to observe a difference in dRnh1-GFP focus-positive cells between the 2 mutants (Supplementary Fig. 2e). A small percentage of cells displaying dRnh1-GFP foci was observed in both strains even in the absence of BED (Supplementary Fig. 2e), consistent with Western blot analysis that indicates low-level dRnh1-GFP expression in the absence of induction (Supplementary Fig. 2a). Percentage of focus-positive cells rapidly increased with medium induction levels (0.5–1 nM BED) and peaked at ≥2 nM BED (Supplementary Fig. 2e). BED concentrations ≥2 nM however also increased background nuclear fluorescence and therefore made scoring live cells for discreet foci more difficult. Possible deleterious effects of dRnh1-GFP expression on cell growth waere also examined. Growth rate measurements of our dRnh1-GFP–expressing strains indicated no reduction in cell growth at or below 0.5 nM BED and, but a slight growth rate reduction was observed at 2 nM (Supplementary Fig. 3).

To standardize our assay conditions, we concluded that a 4-h induction with 0.5–2 nM BED was optimal for detecting changes in R-loop levels. The 0.5-nM BED was used primarily when scoring live cells for dRnh1-GFP foci because of its lack of growth defect (Supplementary Fig. 3) and induction of an easily scorable and significant difference in the percentage of focus-positive cells between the *rnh1Δ* and *rnh1Δ rnh201Δ* mutants (Supplementary Fig. 2e). The 2-nM BED was used to more rigorously induce dRnh1-GFP expression to better capture representative images of cells. Specific BED concentration and treatment times used below are listed in the relevant figure legend.

### Evidence that the dRNh1-GFP reporter protein folds properly and binds nucleic acid

Although dRnh1-GFP focus characteristics and levels plausibly track with expected R-loop formation, an N-terminus-tagged prototype of the dRnh1 reporter (Ivy-dRnh1) (Slubowski *et al*. 2015) generated nuclear foci that proved artifactual. In contrast to dRnh1-GFP foci, the Ivy-dRNH1 foci were large and formed independently of endogenous *RNH1* and the inclusion of the D264N mutation (Supplementary Fig. 4a and b). As demonstrated below, Ivy-dRnh1 “foci” in fact correspond to reporter localization into the Intranuclear Quality Control Compartment (INQ; Supplementary Fig. 4), a disposal site for misfolded nuclear proteins (Borgert *et al*. 2022).

Motivated by these cautionary results, we tested dRNH1-GFP foci for INQ localization in 2 ways. First, using an established cytological reporter for the INQ compartment [VHL-mCherry (Mathew *et al*. 2017)], we found that Ivy-dRnh1 colocalizes to INQ, while dRnh1-GFP does not (Supplementary Fig. 4a). Second, as localization to the INQ compartment depends upon the *BTN2* aggregase (Borgert *et al*. 2022), we tested the effect that a *BTN2* deletion has on focus formation and found that *btn2Δ* eliminates Ivy-dRnh1 foci but not dRnh1-GFP foci. (Supplementary Fig. 4c). These data suggest that Rnh1 is unable to tolerate an N-terminus tag, hence the aggregation of Ivy-dRnh1 into the INQ compartment. In contrast, the C-terminus-tagged dRnh1-GFP foci are unlikely the result of protein misfolding or aggregation.

**Fig. 2.**
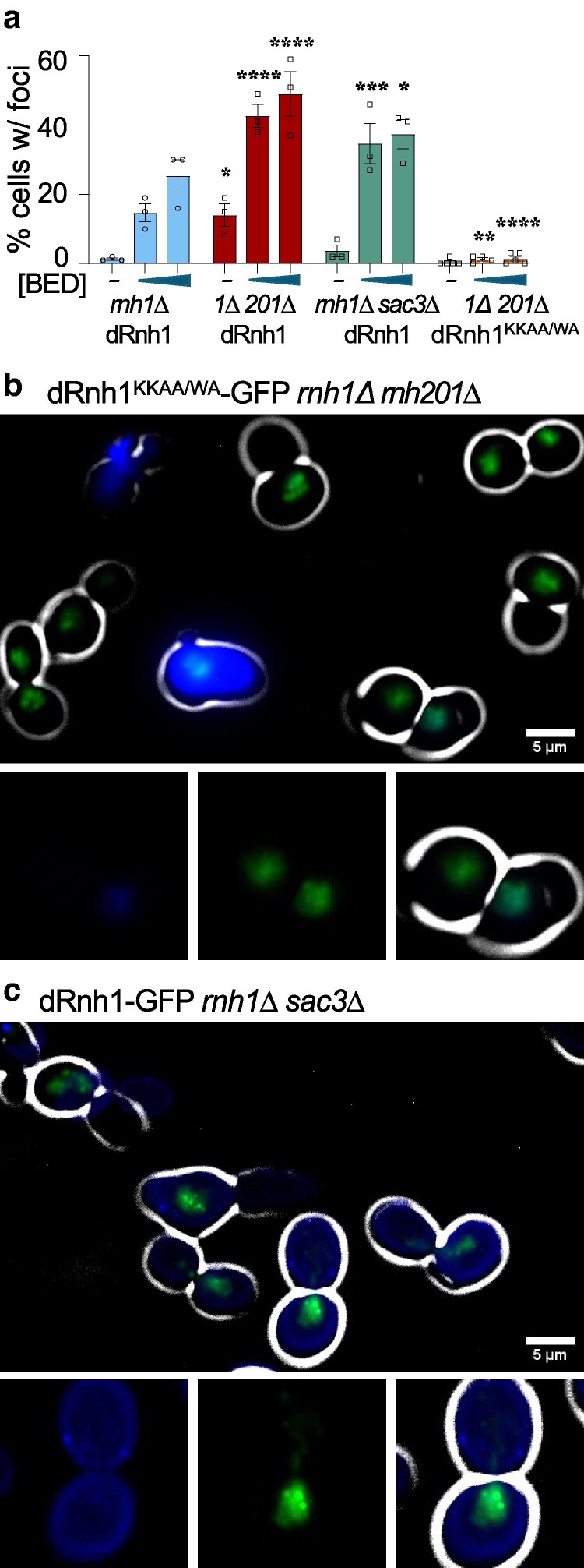
dRnh1-GFP foci are dependent on R-loop binding and can detect changes in R-loops. a) Percentage of live cell population displaying at least one discreet dRnh1-GFP focus after a 4-h incubation in either 0, 0.5, or 1 nM BED. Asterisks denote a statistically significant difference between a given strain/BED treatment and the *rnh1Δ* mutant at the same BED concentration. *rnh1Δ rnh201Δ* strain is denoted by the abbreviation *1Δ 201Δ*. b and c) Representative images of live cells treated with 2 nM BED for 4 h. DAPI-stained DNA is shown in blue, Rhn1-GFP reporters in green, and cell bright field–derived cell walls in white. b) *rnh1Δ rnh201Δ* strain expressing DNA binding–deficient dRnh1^KKAA/WA^-GFP reporter. c) *rnh1Δ sac3Δ* strain expressing dRnh1-GFP.

We investigated if dRnh1-GFP focus formation depends upon nucleic acid binding. Previous structural and biochemical analysis of the human RNase H1 protein demonstrated that 3 critical residues in its N-terminal hybrid-binding domain are needed for R-loop binding (Nowotny *et al*. 2008) and that changing these residues to alanines reduces apparent activity without reducing protein stability (Gaidamakov *et al*. 2005). These residues are conserved in *S. cerevisiae* (W22, K38, and K39) (Supplementary Fig. 1). To test the roles of these residues in focus formation, we generated the corresponding alanine substitution alleles both individually and in combination in the dRnh1-GFP reporter. Western blot analysis indicated that the resulting quadruple mutation (dRnh1^KKAA/WA^-GFP) expressed at levels similar to the standard dRnh1 reporter without displaying a ladder of degradation products (Supplementary Fig. 5a and b). Though dRnh1^KKAA^-GFP, dRnh1^WA^-GFP, and dRnh1^KKAA/WA^-GFP all localized inside the nuclei of live *rnh1Δ* cells, no discreet foci were observed in the *rnh1Δ* mutant (Supplementary Fig. 5c), and foci in *rnh1Δ rnh201Δ* were exceedingly rare (Fig. 2a and b). Together, these data suggest that dRnh1-GFP folds efficiently, and focus formation depends upon the Rnh1 hybrid-binding domain.

### Mutations that increase R-loop levels also increase dRnh1-GFP focus-positive cells

To test assay sensitivity, we next asked whether dRnh1-GFP can detect differences among mutants that were previously shown to increase R-loops. We assayed foci in the *rnh1Δ rnh201Δ* strain, as this mutant combination has previously been shown to increase R-loops compared with a *rnh1Δ* mutant alone (Wahba *et al*. 2011). We found that the number of focus-positive cells was significantly higher in the *rnh1Δ rnh201Δ* double mutant (Figs. 1d and 2a). In addition, we examined a deletion of *SAC3*, a component of the mRNA exporting TREX (TRanscription-EXport) complex, that has previously been shown to increase R-loop levels (Gonzalez-Aguilera *et al*. 2008). dRnh1-GFP expression was induced using 0.5 and 1 nM BED in a *rnh1Δ sac3Δ* double mutant (Fig. 2a and c). Though not as dramatic as in *rnh1Δ rnh201Δ* cells, the *rnh1Δ sac3Δ* strain displayed a 2-fold increase in focus-positive cells compared with *rnh1Δ* alone (Fig. 2a). These results validate that the dRnh1-GFP reporter has sufficient sensitivity to discriminate between a different R-loop levels.

### dRnh1-GFP is a suitable reporter for drug screening

Given the possible involvement of R-loops in human disease (*Introduction*), identifying small molecules that modulate R-loop levels may prove valuable. To evaluate the utility of the dRnh1- GFP reporter in small molecule screening, we test below well-characterized drugs that either cause DNA damage or more specifically target DNA replication. Their effect on dRnh1-GFP focus formation was studied in both the *rnh1Δ* and the more sensitized *rnh1Δ rnh201Δ* strains using previously established and physiologically relevant concentrations (Ngo *et al*. 2019; Sanders *et al*. 2022).

**Fig. 3.**
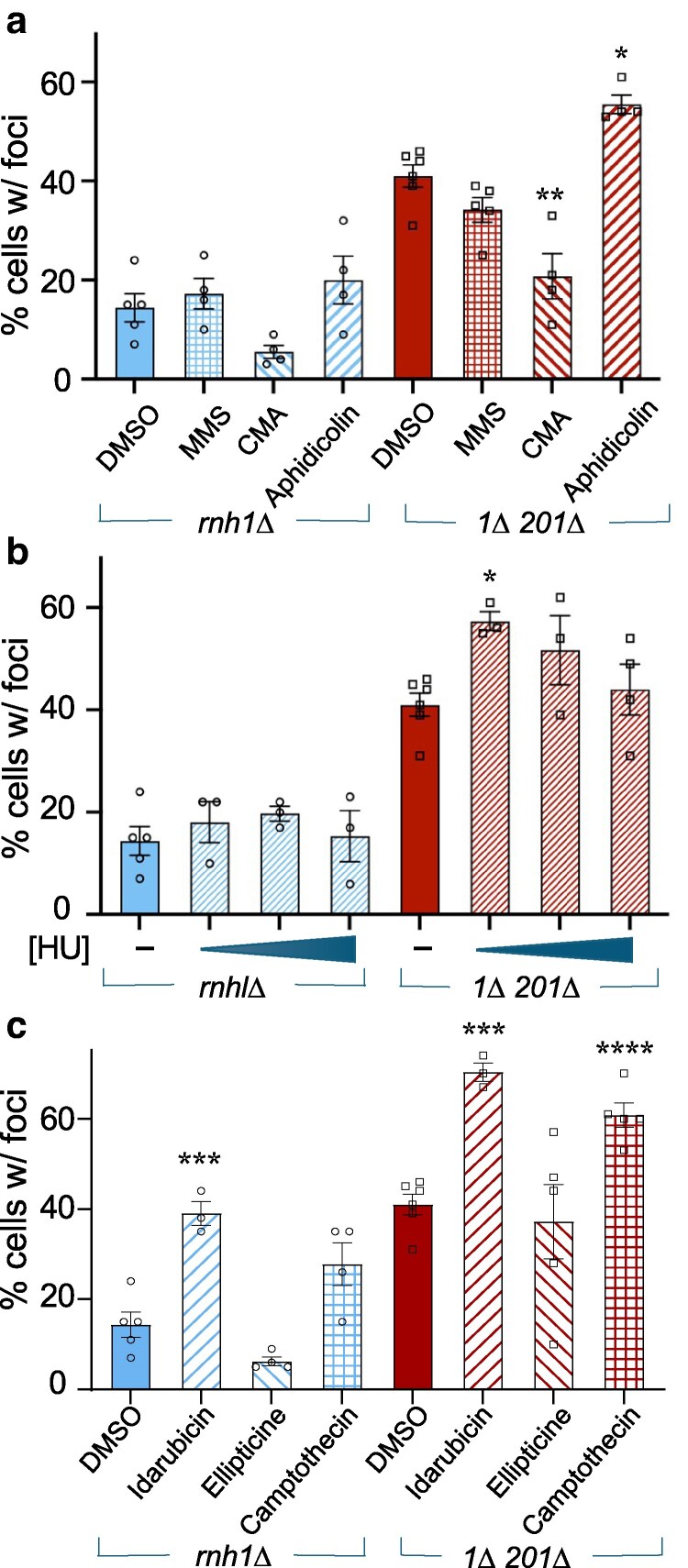
Percentage of cells displaying dRnh1-GFP foci changes in response to drug treatments. *rnh1Δ* or *Δrnh1Δ rnh201Δ* strains were simultaneously treated with 0.5 nM BED and the indicated drug for 4 h. Live cells were harvested and scored for the percentage of cells in the population displaying at least one discreet dRnh1-GFP focus. Asterisks indicate a statistically significant increase over DMSO-treated cells. a) Cells were treated with 0.5% DMSO, 0.03% MMS, 125 μM aphidicolin, or 250μ M CMA. b) Cells were treated with 25 mM, 50 mM, or 200 mM HU. c) Cells were treated with 260 μM camptothecin, 100 μM ellipticine, or 10 μM idarubicin.

To test if dRnh1-GFP foci levels are stimulated by nonspecific DNA damage, we tested the effect of the DNA alkalization agent methane methyl sulfonate (MMS) on focus development. MMS treatment causes extensive DNA methylation, leading to DNA double-strand break formation (Vijayraghavan *et al*. 2016). MMS had no effect on the percentage of dRnh1-GFP focus-positive cells at the concentration tested (Fig. 3a), suggesting that foci do not form as a casual consequence of alkalization damage.

In contrast to MMS, the percentage of *rnh1Δ rnh201Δ* cells displaying dRnh1-GFP foci increased when treated with the DNA polymerase inhibitor aphidicolin (Fig. 3a) This result is consistent with a previous study showing that this drug increases R-loop formation (Andrs *et al*. 2023). Interestingly, the putative Mcm2-7 helicase inhibitor N-methyl-β-carboline-3-carboxamide (CMA) shown to slow or halt the cell cycle in S-phase (Sanders *et al*. 2022) decreases the percentage of focus-positive *rnh1Δ rnh201Δ* cells (Fig. 3a). Taken at face-value, this indicates that replicative helicase inhibition may prevent R-loop formation instead of exacerbating it, but further investigation is necessary to determine the possible biological relevance.

Hydroxyurea (HU) causes replication fork arrest and collapse through the inhibition of ribonucleotide reductase and subsequent dNTP depletion (Andrs *et al*. 2023). In human cells, HU can cause R-loop-dependent replication fork arrest only at low HU concentrations that are still permissive for dNTP synthesis and DNA replication (Andrs *et al*. 2023). This finding has led to a general model wherein low HU levels exacerbate transcription-replication conflicts/R-loops by destabilizing and modestly slowing replication forks (Andrs *et al*. 2023). In contrast, high HU levels block both replication and transcription-replication conflicts and thus do not stimulate R-loop formation (Andrs *et al*. 2023). Assaying focus formation over a range of HU concentrations, we also found the percentage of focus-positive *rnh1Δ rnh201Δ* cells increased at low HU concentrations (25 mM) (Fig. 3b), while higher concentrations (50–200 mM) caused no statistical change.

Both topoisomerases I and II are well-studied attenuators of R-loop formation (Patel *et al*. 2022). Consistent with these findings, *rnh1Δ* and *rnh1Δ rnh201Δ* cells displayed a 2-fold increase in focus-positive cells when treated with the topo I inhibitor camptothecin, our most significant finding of any drug tested (Fig. 3c). We also tested 2 topo 2 inhibitors with differing mechanisms of action. Ellipticine prevents topo 2 from interacting with DNA (Cline *et al*. 1997). In contrast, idarubicin acts as a poison and traps topo 2 in complex with DNA (Hande 2008). We found that the topo 2 inhibitor ellipticine (Fig. 3c) had no effect on dRnh1-GFP focus-positive cells, but idarubicin increases the percentage of foci (Fig. 3c).

In summary, we find that drugs previously reported to modulate R-loop levels in human cells also similarly modulate dRnh1-GFP foci levels in budding yeast. This lends strength to dRnh1-GFP's efficacy as tool to identify drugs that increase R-loops in live cells.

### Characterizing dRnh1-GFP and R-loops in chromatin spreads

**Fig. 4.**
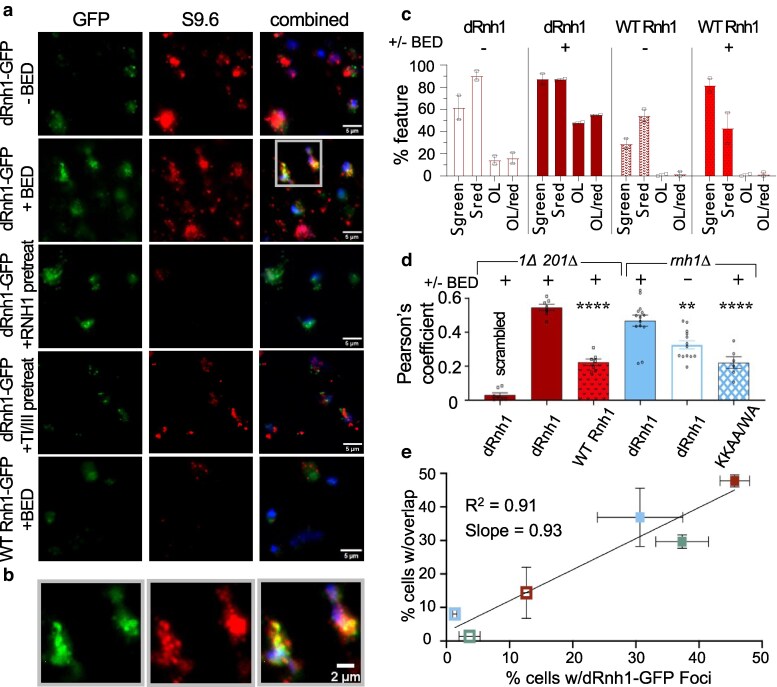
dRnh1-GFP colocalizes with R-loops. a) Representative images of chromatin spread IF. *rnh1Δ rnh201Δ* cells containing the indicated GFP reporter constructs were grown for 4 h in ±2 nM BED and harvested for S9.6 IF. RNase H/TI/TIII pretreatments were conducted as described in *Materials and Methods*. GFP fluorescence is indicated in green, R-loops as defined by S9.6 antibody in red, and DAPI-stained nucleus in blue. b) Magnified image identical to the inset defined in a). c) Results of visual scoring of IF images. *rnh1Δ rnh201Δ* strain was scored for the presence of dRnh1-GFP fluorescence (Σ green), S9.6 fluorescence (Σ red), and overlap between dRnh1-GFP/S9.6 signal (%OL). Results of this scoring are represented as a percentage of cells in the population displaying a given feature and the percentage of cells displaying S9.6 signal that also display overlapping dRnh1-GFP signal (%OL/red). d) Pearson's colocalization coefficient between dRnh1-GFP and S9.6 antibody derived from chromatin spread IF. Asterisks indicate a significant difference between the indicated strain and the *rnh1Δ* strain treated with 2 nM BED. e) Linear regression comparing percentage of dRnh1-GFP–positive cells in live cell analysis with the percentage of nuclei displaying dRNh1-GFP/S9.6 overlap in parallel chromatin spreads. Open symbols, no BED; closed symbols, +2 nM BED. Blue *rnh1Δ,* green *rnh1Δ sac3Δ*, red *rnh1Δ rnh201Δ*. Results plotted from data are shown in Figs. 2a and 4c and Supplementary Fig. 6d.

To directly compare dRnh1-GFP and R-loops in the same nuclei, we used an established chromatin spread assay designed to visualize R-loops using S9.6 IF (Wahba *et al*. 2011) and adapted the analysis (Sup Methods) to score for dRnh1-GFP and S9.6 signals. This protocol gently lyses the cell prior to fixation (Wahba *et al*. 2011), enlarges and flattens the nucleus into a single focal plane (Wahba *et al*. 2011; Vijayraghavan *et al*. 2016), and largely eliminates S9.6 cross-reacting dsRNA (Crossley *et al*. 2021). The dRnh1-GFP reporter survives fixation and was directly visualized with high efficiency (green), while R-loops were visualized using a fluorescent secondary antibody (red) (Fig. 4a; Supplementary Fig. 6a). The DNA stain DAPI was included in the assay to identify nuclei; only red (R-loops, S9.6) or green (dRnh1-GFP) signals within ∼1 µm of the DAPI staining were considered valid. Following appropriate thresholding to remove nonspecific fluorescence, we find that the green signal is specific for dRnh1-GFP as uninduced cells had reduced green signal (Fig. 4a). Control experiments indicated however that the red signal corresponded to a mixture of R-loop and double-stranded RNA species as had been previously observed (Crossley *et al*. 2021). Pretreatment of slides with commercial RNase H prior to addition of the S9.6 antibody overall eliminated >50% of the red signal (Fig. 4a; Supplementary Fig. 6b). Slides were also pretreated with a mixture of RNaseT1 (single-strand specific) and RNAse III (double-strand specific), a treatment that reduced the overall level of S9.6 signal by ∼30% (Supplementary Fig. 6b). The S9.6 signal however is RNA specific, as pretreatment of slides with all 3 nucleases completely eliminated the red signal (Fig. 4a; Supplementary Fig. 6b). The relationship between the different RNA species and the green dRnh1-GFP signal will be discussed below.

Chromatin spreads are more sensitive but considerably more complex and difficult to score compared with live-cell imaging. Unlike live cell imaging in which nuclei are condensed and possess a 3D structure (Fig. 1b and c), the enlarged and “flattened” nuclei characteristic of chromatin spreads allows for viewing of red and green signals at an effectively greater resolution. This greater resolution allows one to observe more complex phenotypes in nuclei. dRnh1-GFP foci are readily observable in the chromatin spreads, but many nuclei feature secondary dRnh1-GFP signals that cannot be easily differentiated in live-cell imaging (Fig. 4a; Supplementary Fig. 6a top) because of the compaction and 3D structure of the nucleus. The red S9.6 signal is generally more abundant than dRnh1-GFP signal (see *Discussion*) and can be observed as either individual spots or as dense clusters of spots, with clusters being more abundant in *rnh1Δ rnh201Δ* cells (compare Fig. 4a; Supplementary Fig. 6a). dRnh1-GFP signal can colocalize however with either the clusters or individual S9.6 spots (Fig. 4a; Supplementary Fig. 6a top), which necessitates careful analysis of cells to identify examples of overlap. Moreover, the data interpretation depends on whether they are presented as total fluorescence or as the fraction of cells that contain a nuclear signal.

### dRnh1-GFP colocalizes with R-loops in chromatin spreads

Simple visual comparison of micrographs from *rnh1Δ* and *rnh1Δ rnh201Δ* chromatin spreads indicate significant spatial overlap between the S9.6 and dRnh1-GFP signals (Fig. 4a and b; Supplementary Fig. 6a). The overlap extent is highly dependent upon the same parameters as dRnh1-GFP foci in living cells; signal overlap is highest in chromatin spreads containing the dRNh1-GFP reporter, while conspicuously absent in spreads expressing either the wild-type RNH1-GFP or dRnh1^KKAA/WA^-GFP reporters (Fig. 4a; Supplementary Fig. 6a discussed below). The S9.6 signal that overlaps dRnh1-GFP appears to be predominantly R-loops, as preincubation of the slides with RNase H eliminates >75% of the overlap signal (Fig. 4a; Supplementary Fig. 6c). In contrast, pretreatment of the slides with RNase T1/III caused no reduction in overlap between the red and green signals (Fig. 4a; Supplementary Fig. 6c). As such signal overlap is observed for both S9.6 spots and clusters, we will simplify the data below by combining both classes and considering them together. Two approaches were used for quantification of red/green signal overlap. First, each DAPI-staining nucleus was visually scored for the presence of S9.6 signal, presence of dRnh1-GFP signal, and whether these 2 signals spatially overlap and shared a similar cytological pattern (e.g. Fig. 4b). Results are presented as percentage of cells with the listed feature.

About 90% of *rnh1Δ rnh201Δ* nuclei display some level of S9.6 signal, regardless of dRnh1-GFP induction (Fig. 4c), indicating that reporter expression does not substantially change R-loop levels. In addition, ∼60% of nuclei contain some dRnh1-GFP signal in the absence of BED. Nevertheless, only ∼15% of the S9.6-positive nuclei demonstrate overlap with dRnh1-GFP (Fig. 4c). Following 2 nM BED induction of dRnh1-GFP, green fluorescence/nuclei considerably increases (90%) along with a 3.6-fold concomitant increase (55%) in green/red colocalization in S9.6-positive nuclei (Fig. 4c). Note that in this condition, colocalizing signals are not specific to either spots or clusters and occur with nearly equal frequency in both groups. Confirming the qualitative inspection, *rnh1Δ rnh201Δ* cells expressing the wild-type Rnh1-GFP reporter reduces S9.6-positive nuclei 2-fold even in the absence of BED induction (∼45%, Fig. 4c), confirming that Rnh1-GFP has functional RNaseH activity. Induction of wild-type Rnh1-GFP importantly results in extremely little overlap between GFP and S9.6 signals (∼5%), confirming that colocalization of the 2 signals is dependent on the inactivation of Rnh1's nuclease domain (Fig. 4c). Moreover, although expression of the wild-type construct eliminates dRnh1-GFP foci in living cells (Fig. 1d), expression of the wild-type construct in the *rnh1Δ rnh201Δ* strain reduces but does not eliminate R-loops, supporting our earlier finding that focus formation is eliminated in a *RNH1* background because it out-competes the reporter, not because R-loops are absent. The visual scoring indicates overall that the dRnh1-GFP and S9.6 signals significantly colocalize.

We calculated the Pearson's correlation coefficient (*Materials and Methods*) to obtain an alternative and more quantitative metric for colocalization. This value represents the correlation between the 2 colors (red and green) on a pixel-by-pixel basis, with a coefficient of +1 corresponding to perfect correlation and −1 corresponding to perfect anticorrelation. For reference, following randomization of the S9.6 signal in one of our micrographs, the calculated Pearson's coefficient is ∼0 (Fig. 4d “scrambled”); thus, confirming that background signal is not contributing to an artificial increase in the correlation value and setting a comparison baseline for our below analysis.

In the *rnh1Δ* strain containing the dRnh1-GFP reporter and lacking BED induction, the green and red signals weakly correlate (∼0.325, Fig. 4d). Following BED induction, the Pearson's coefficient significantly increased to ∼0.467 for this strain. This value increased further to 0.545 for the BED-induced expression of dRnh1-GFP in the *rnh1Δ rnh201Δ* strain (Fig. 4d). In control strains expressing either the dRnh1^KKAA/WA^-GFP or wild-type RNH1-GFP reporter, the Pearson's coefficient indicated negligible overlap, with both values averaging ∼0.23 (Fig. 4d; Supplementary Fig. 6a and d).

Chromatin spreads were additionally prepared and analyzed when compared with our other experiments: *rnh1Δ sac3Δ* and the *rnh1Δ*  *rnh2*01*Δ* mutants exposed to various replication inhibitors tested in Fig. 3. Though there was no significant change in Pearson's correlation coefficient between the *rnh1Δ* and *rnh1Δ sac3Δ* mutants, the resultant coefficients calculated for *rnh1Δ*  *rnh2*01*Δ* cells treated with drugs largely mirrored the results of observing dRnh1-GFP focus-positive cells in the live-cell assay (compare Fig. 3 with Supplementary Fig. 6e). Drugs that caused significant increases in dRnh1-GFP focus-positive cells (aphidicolin, HU, and idarubicin) also caused near-significant increases in the Pearson's coefficient for dRnh1-GFP/S9.6 overlap (Supplementary Fig. 6e).

Both visual scoring and calculation of the Pearson's coefficient indicate that dRnh1-GFP foci shows moderate colocalization with S9.6-defined R-loops. However, how well does our live-cell dRnh1-GFP focus assay correlate with the chromatin spread analysis? Among our experiments containing direct comparable live cell and chromatin spread data, we compared the results from these 2 different assays using linear regression. The analysis shows a significant positive correlation (*R*^2^ = 0.91) between the level of dRnh1-GFP foci from live cell analysis and the percentage of nuclei containing green/red overlap from the chromatin spread assay (Fig. 4e). Moreover, given that the slope of the regression is nearly 1.0 (slope = 0.93) this correlation is one-to-one—i.e. on average, a cell containing a dRnh1-GFP focus in the live cell assay corresponds to a nucleus in the chromatin spread assay with an overlap between green and red. If one defines true R-loops as those that are coordinately identified by both dRnh1-GFP and S9.6 antibody, the live cell assay is excellently predictive of R-loop levels.

## Discussion

Our data support the use of dRnh1-GFP as a fast and effective alternative to the S9.6-mediated cytological assay in both living and fixed cells of *S. cerevisiae*. The dRnh1-GFP reporter colocalizes with R-loops (Fig. 4), and our live-cell assay generates an easily scorable phenotype (Fig. 1) that reliably and reproducibly quantifies R-loop levels. dRnh1-GFP also detects changes in R-loops levels in pilot genetic (Fig. 2) and drug screens (Fig. 3). The overall simplicity of yeast as a model organism significantly decreases the cost, complexity, and time needed to screen cells for R-loops. S9.6-based IF experiments that previously required 3–5 days of work and follow-up analysis (Wahba *et al*. 2011) can now be completed in a single day using the dRnh1-GFP reporter system. This assay also lends itself to high-throughput screens, which will serve as powerful tools for identifying new genes and drugs associated with R-loop attenuation.

### Sensitivity of dRnh1-GFP compared with S9.6 assays

Though the percentage of dRnh1-GFP–positive and S9.6-positive nuclei were similar in chromatin spreads of BED-induced cells (Fig. 4c), in general we found that total S9.6 signal was much more abundant than dRnh1-GFP signal, especially in the *rnh1Δ rnh201Δ* mutant. The moderate Pearson's correlation coefficients (Fig. 4d) derived from chromatin spreads also indicate that a subset of dRnh1-GFP and S9.6 signal do not colocalize. Given that the data support that dRnh1-GFP is detecting R-loops, what is the most likely reason for this imperfect colocalization? Examination of these 2 reporters’ affinity for R-loops and potential interfering dsRNA may provide a reasonable explanation. S9.6 has not only an exceptionally high affinity for R-loops (*K_d_* = 0.6 nM) but also a high affinity for dsRNA (*K_d_* = 2.7 nM) (Crossley *et al*. 2021). In comparison, human Rnh1's hybrid-binding domain has a lower affinity for R-loops (*K_d_*∼200 nM) but overall is much less likely to bind to dsRNA (*K_d_* = 4.9 µM) (Nowotny *et al*. 2008). The difference in sensitivity/specificity of these 2 reporters is possibly one reason for differences in colocalization. Additionally, there is the possibility that S9.6 detects a wider array of R-loops compared to dRnh1-GFP. Genomic studies of mammalian-derived dRNH1 and S9.6 propose that dRNH1 primarily detects R-loops at transcription start sites, while S9.6 detects R-loops across the entirety of a gene body (Miller *et al*. 2022). dRnh1-GFP is therefore likely the less sensitive but much more specific reporter compared to S9.6. However, dRnh1-GFP's lower affinity for R-loops may be overcome and colocalization may improve if dRnh1-GFP is expressed at higher levels than what was tested in this study.

### Implications of Rnh1 mechanism in *S. cerevisiae*

During our studies, we were initially concerned that the dRnh1 mutant would be a dominant allele and artificially elevate R-loop levels by trapping and preventing their repair. Fortunately, the allele appears to act recessively and does not seem to substantially alter R-loop levels (compare Σ red +/− BED in Fig. 4c). However, being recessive, it became necessary to remove the endogenous RNH1 gene to facilitate reporter function.

Though different from expectations, the recessive nature of dRnh1 is consistent with other discoveries on the nature of Rnh1's mechanism (thoroughly reviewed in the study by Cerritelli and Crouch 2009). Rnh1's nuclease domain and hybrid-binding domain are connected by a flexible connection domain. This allows for Rnh1 to assume different conformations and cut RNA at multiple different locations, while the hybrid-binding domain remains in a fixed location. RNA in R-loops therefore is not completely degraded by Rnh1, but rather cut into multiple smaller pieces that overall have a lower affinity for DNA compared with the complementary strand they are displacing. This competition combined with DNA's stabilization upon reassuming B-form provides the energetic impetus for the removal of the small RNA/DNA hybrids. Wild-type Rnh1 would therefore not require access to the entirety of an R-loop in order to remove it, as even an R-loop that is mostly occupied by dRnh1 could be eventually degraded with a few key cuts by wild-type Rnh1. With Rnh1's mechanism taken into account, it is easy to understand why wild-type Rnh1 can seemingly “out-compete” overexpressed dRnh1-GFP.

The necessity of deleting wild type *RNH1* does lead to some complications. The most obvious one is that all yeast studies utilizing dRnh1-GFP must be conducted in a mutant background, adding extra strain construction steps/limitations and rendering R-loop analysis in a wild-type strain background impossible. Nevertheless, we anticipate that yeast dRnh1-GFP will prove a useful system for a variety of powerful screens and assays.

### dRnh1-GFP focus size and abundance

As noted above, dRnh1-GFP commonly form single foci in living cells, but multiple foci in chromatin spreads. These differences, however, may be more of a visual illusion than a substantiative distinction. From our representative images (Fig. 1b and c), one can see that although dRnh1-GFP often forms one single predominant focus per cell (primary focus), it also forms additional less prominent and sometimes smaller foci (secondary foci). These smaller “secondary foci” are not easily visible when inspecting live cells under the microscope but become visible in micrographs, especially when dRnh1-GFP is induced with higher concentrations of BED. These secondary foci become still more visible in chromatin spreads (Fig. 4a; Supplementary Fig. 6a), most likely because this procedure “flattens” the nucleus and brings every focus into a single plane.

What then distinguishes primary from secondary foci? One possibility is that primary dRnh1-GFP foci correspond to rare large R-loops, while shorter R-loops result in secondary foci or are less discernable from background fluorescence. Alternatively, it is possible that focus prominence represents a specific area of the nucleus where R-loops are prevalent. For example, nucleoli are hotspots for transcription and therefore R-loop formation (Shen *et al*. 2017). Nucleoli can be differentiated from the larger nucleus in chromatin spreads by their close proximity to the nucleus and reduced DAPI staining (Vijayraghavan *et al*. 2016). Our S9.6 IF indeed confirmed that nucleolar R-loops were present in our cell populations (see Supplementary Fig. 6a, *rnh1Δ* dRnh1^KKAA/WA^-GFP for example). Large primary foci could therefore represent nucleolar R-loops, while secondary foci represent R-loops within other regions of the nucleus where transcription is less prevalent.

### R-loop repair factories

Another possible distinction between primary and secondary foci is the number of R-loops that each contain. The ability to visualize bright and discreet nuclear dRnh1-GFP foci using a standard fluorescence microscope implies that there are many copies of dRnh1-GFP localized to a specific area inside the nucleus. Although we do not know the number of dRnh1-GFP molecules present in our foci, careful analysis of Rad52-YFP DNA double-strand break repair foci (Lisby *et al*. 2003) in budding yeast may provide an appropriate proxy. A prior study of Rad52-YFP posited that visible Rad52-YFP repair foci represent 600–2,100 molecules of Rad52-YFP localized to multiple strands of DNA (a “repair factory”) (Lisby *et al*. 2003). Given such precedence, how many R-loops may be present in a dRnh1-GFP focus? As stated earlier, R-loops may range in size from 100 to 2,700 bp (Huang *et al*. 2006; Stolz *et al*. 2019; Malig *et al*. 2020), with the average human R-loop being ∼310 bp (Malig *et al*. 2020) and 70% of *S. cerevisiae* R-loops measuring <500 bp (Wahba *et al*. 2016). Crystal structures of human RNase H1 indicate that it occludes 11–12 bases when bound to DNA (Cerritelli and Crouch 2009; Hyjek *et al*. 2019). Assuming rough equivalence of dRnh1-GFP occupancy and R-loop length between yeast and human Rnh1, the average 310-bp R-loop could be occupied by a maximum of 25–28 molecules of dRnh1-GFP, while rare 1-kb R-loops could theoretically bind 83–90 dRnh1-GFP molecules. Assuming then that a long R-loop averages about 100 GFP molecules and that dRnh1-foci have roughly similar number of GFP molecules as RAD52-YFP foci, this would suggest that each dRnh1-GFP focus represents the colocalization of 6–21 R-loops and corresponds to a repair factory. dRnh1-GFP foci are not simply a product of reporter overexpression, as foci are observed in a small percentage of cells even in the absence of BED induction (Fig. 2a). However, given that the wild type Rnh1 fails to form observable foci (Fig. 1d), it is unclear if colocalization of R-loops is a unique property of *rnh1Δ* strains or ir it also occurs under wild type conditions.

### Wider application of the dRnh1-GFP assay

Despite progress in the study of human cells, previously no techniques existed to study R-loops in live yeast cells, relegating R-loop research almost entirely to low-throughput candidate approaches that mostly focused on human cells. Our dRnh1-GFP reporter opens the door for intriguing possibilities, as yeast are especially easy to maintain and manipulate genetically and a wide variety of techniques are available to study transcription and replication. Yeast studies have provided insights into human disease, particularly diseases associated with DNA replication, DNA damage, and DNA repair (Botstein and Fink 2011). dRnh1-GFP extends the possibility of vital disease research to *S. cerevisiae*. Moreover, combined with the experimental tools available in yeast, our assay is sufficiently simple to be automated for use in either large-scale high-content drug discovery or unbiased forward genetic screen.

## Supplementary Material

jkaf072_Supplementary_Data

## Data Availability

Strains and plasmids are available upon request. The authors affirm that all data necessary for confirming the conclusions of the article are present within the article, figures, and tables. Supplemental material available at G3 online.
