# Supplementary material for: An improved cytological assay for R-loop detection in Saccharomyces cerevisiae utilizing a catalytically inactive RNase H
Source: G3 (Bethesda). 2025 Apr 10;15(6):jkaf072. doi: 10.1093/g3journal/jkaf072 (PMC12134985; doi:10.1093/g3journal/jkaf072)
Supplement: jkaf072_Supplementary_Data [file jkaf072_supplementary_data.zip › Figure_S1_G3-2024-405428.pdf]

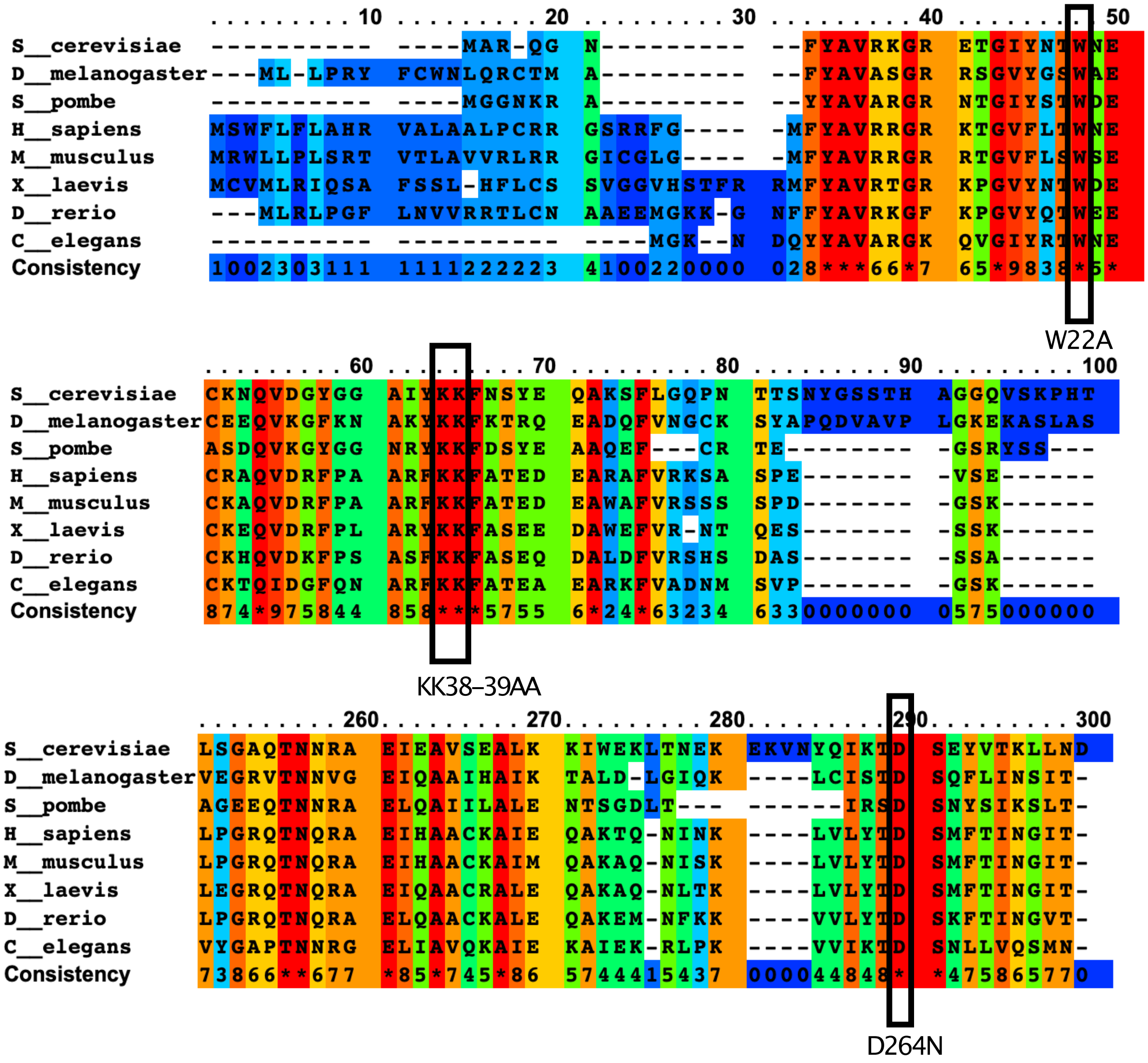

Fig. S1. PRALINE-derived multiple sequence alignment of RNase H1 (/www.ibi.vu.nl/programs/pralinewww/). Key conserved residues mutated in this study are bound by black rectangles and numbering corresponds to the yeast Rnh1. Mutations W22A, K38A, and K39A are within the DNA binding domain and disrupt Rnh1's ability to bind R-loops. Mutation D264N eliminates Rnh1's nuclease activity.
