# Supplementary material for: An improved cytological assay for R-loop detection in Saccharomyces cerevisiae utilizing a catalytically inactive RNase H
Source: G3 (Bethesda). 2025 Apr 10;15(6):jkaf072. doi: 10.1093/g3journal/jkaf072 (PMC12134985; doi:10.1093/g3journal/jkaf072)
Supplement: jkaf072_Supplementary_Data [file jkaf072_supplementary_data.zip › Figure_S2_G3-2024-405428.pdf]

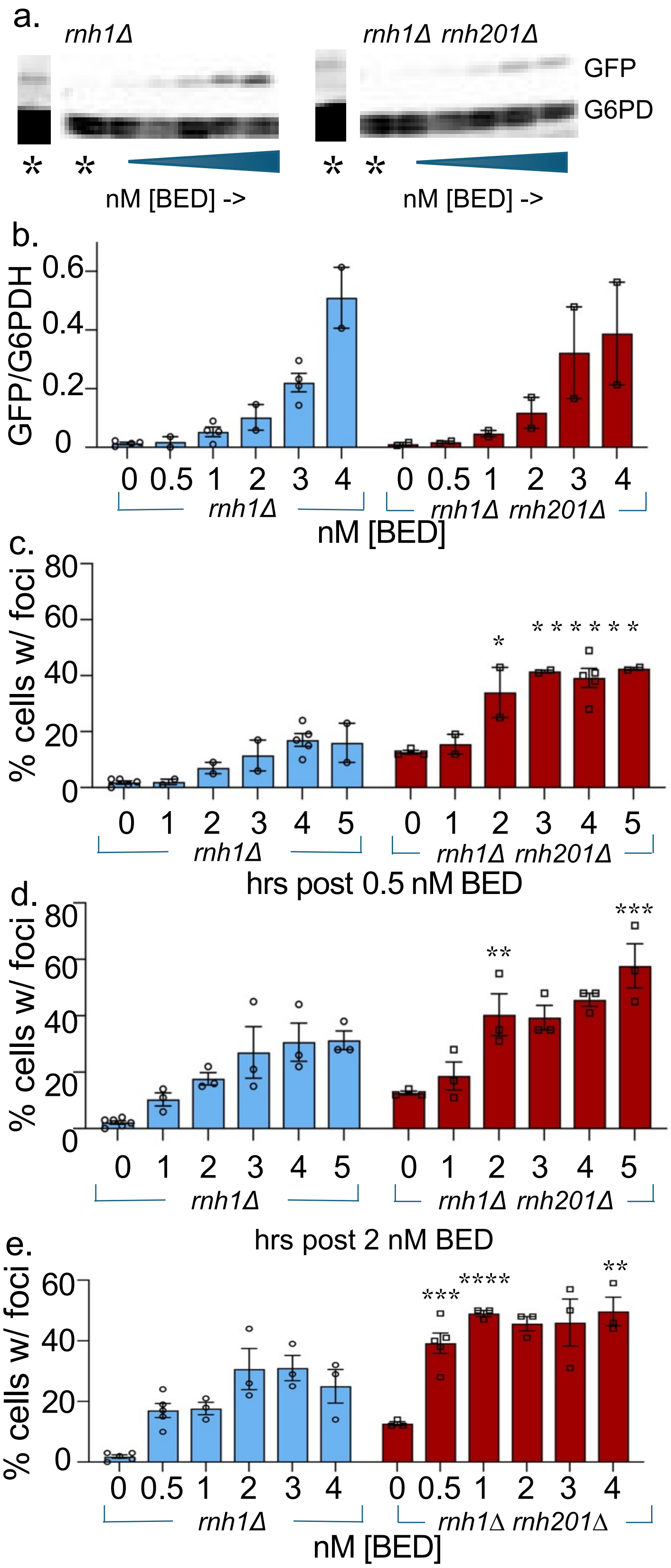

Fig. S2. (a) Representative Western blot of *rnh1Δ* or *rnh1Δ rnh201Δ* strains expressing dRnh1-GFP. Cells were induced for 4 hours with 0, 0.5, 1, 2, or 4nM BED. Inset indicated by the asterisk depicts a higher exposure of lane 1 (uninduced cells). (b) Quantitation of Western blots depicted in a. Average pixel intensity of GFP band/average pixel intensity of G6PDH. (c-d) *rnh1Δ* or *rnh1Δ rnh201Δ* cells were treated with 0.5nM BED (c) or 2nM BED (d) and samples were taken over a 5-hour period. Percentage of live cells displaying at least one discreet dRnh1-GFP focus was recorded. Asterisks represent a statistically significant difference in % focus levels between the two strains at the indicated time point. (e) The same strains were grown in a titration of BED for 4 hours. Percentage of live cells displaying at least one discreet dRnh1-GFP focus was recorded. Asterisks indicate statistically significant difference in % focus-positive cells between the two strains at the indicated [BED].
