# Supplementary material for: An improved cytological assay for R-loop detection in Saccharomyces cerevisiae utilizing a catalytically inactive RNase H
Source: G3 (Bethesda). 2025 Apr 10;15(6):jkaf072. doi: 10.1093/g3journal/jkaf072 (PMC12134985; doi:10.1093/g3journal/jkaf072)
Supplement: jkaf072_Supplementary_Data [file jkaf072_supplementary_data.zip › Figure_S3_G3-2024-405428.pdf]

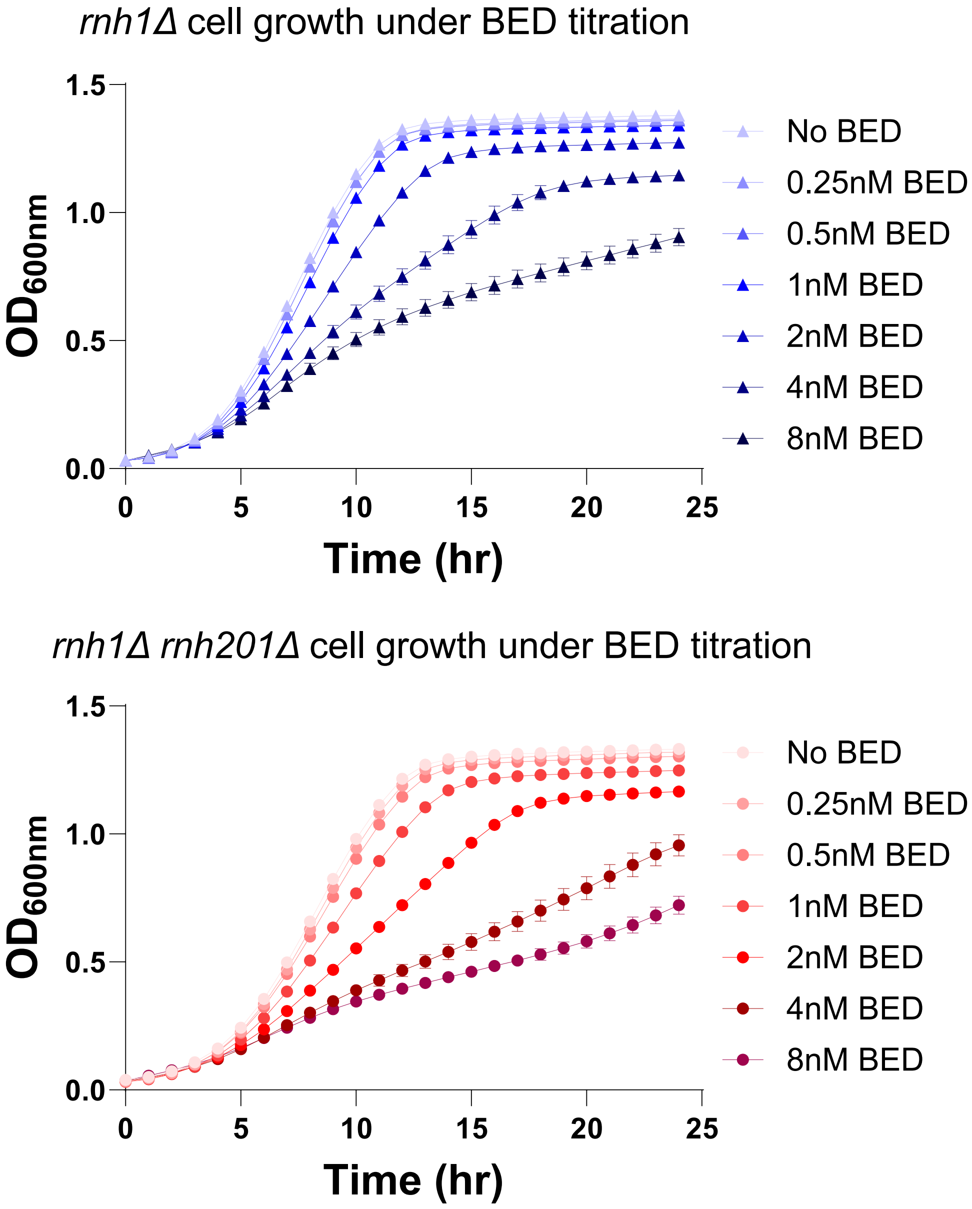

Fig. S3. 24-hour growth curves of *rnh1Δ* and *rnh1Δ rnh201Δ* strains exposed to a titration of BED. Cells were grown at 30°C in YPD with continuous agitation. OD_{600nm} was recorded hourly.
