# Supplementary material for: An improved cytological assay for R-loop detection in Saccharomyces cerevisiae utilizing a catalytically inactive RNase H
Source: G3 (Bethesda). 2025 Apr 10;15(6):jkaf072. doi: 10.1093/g3journal/jkaf072 (PMC12134985; doi:10.1093/g3journal/jkaf072)
Supplement: jkaf072_Supplementary_Data [file jkaf072_supplementary_data.zip › Figure_S4_G3-2024-405428.pdf]

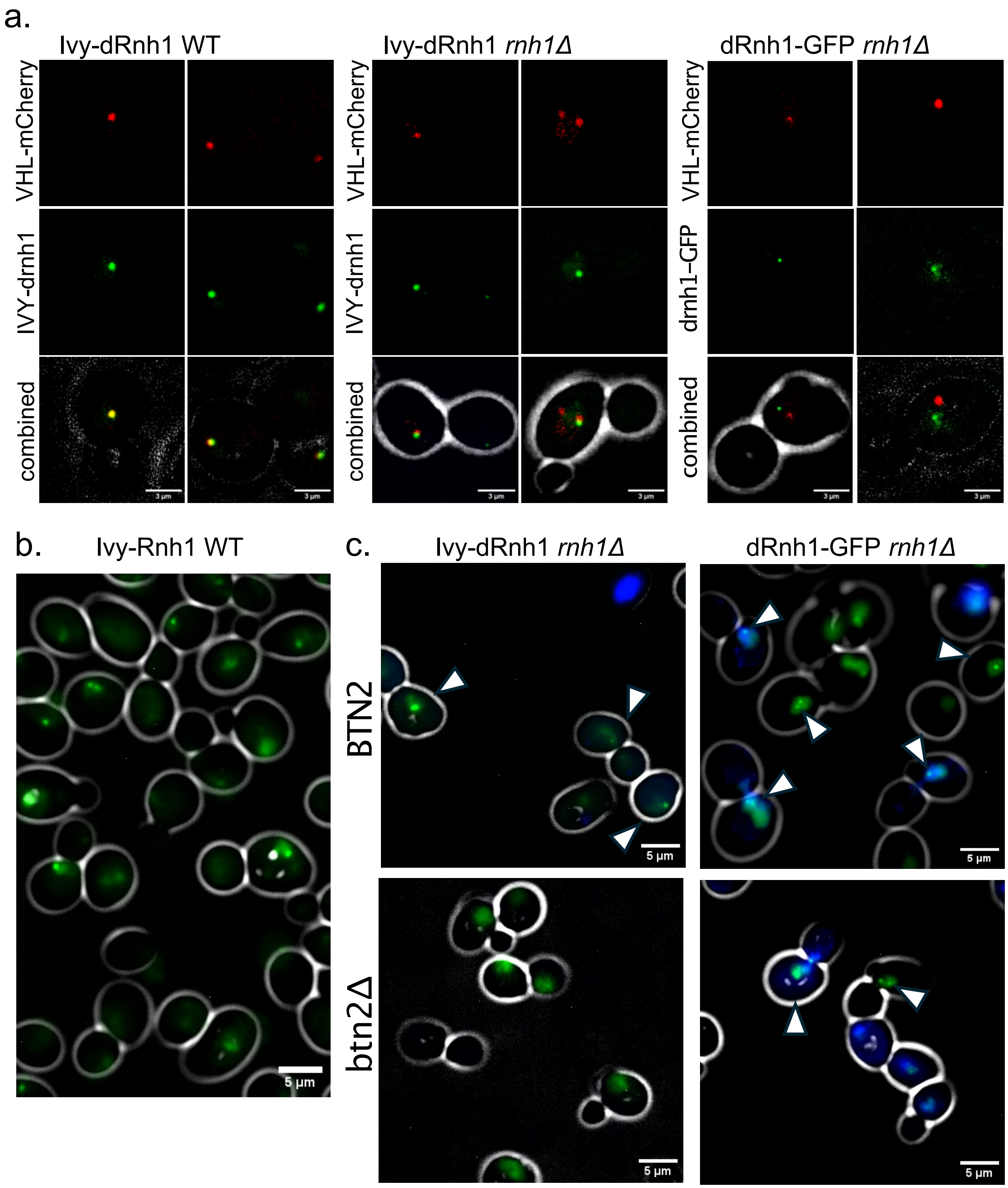

Fig. S4. dRnh1-GFP does not aggregate in protein quality control compartment. (a) Representative images of wild type or *rnh1Δ* strains induced to express either Ivy-dRnh1 or dRnh1-GFP with 2nM BED for 4 hours at 37° C. Cells simultaneously expressed a VHL-mCherry (red) reporter for labelling of INQ quality control compartment. (b) Representative image of wild type cells expressing Ivy-Rnh1. (c) Representative images of *rnh1Δ* strains induced to express either N-terminus-tagged Ivy-dRnh1 or C-terminus-tagged dRnh1-GFP with 2nM BED for 4 hours. BTN2 quality control sequestrase deleted in indicated strains. Ivy-dRnh1 and dRnh1-GFP are indicated in green with the DAPI-stained nuclei indicated in blue. Cells containing discreet dRnh1 foci are indicated with white arrowheads.
