# Supplementary material for: An improved cytological assay for R-loop detection in Saccharomyces cerevisiae utilizing a catalytically inactive RNase H
Source: G3 (Bethesda). 2025 Apr 10;15(6):jkaf072. doi: 10.1093/g3journal/jkaf072 (PMC12134985; doi:10.1093/g3journal/jkaf072)
Supplement: jkaf072_Supplementary_Data [file jkaf072_supplementary_data.zip › Figure_S5_G3-2024-405428.pdf]

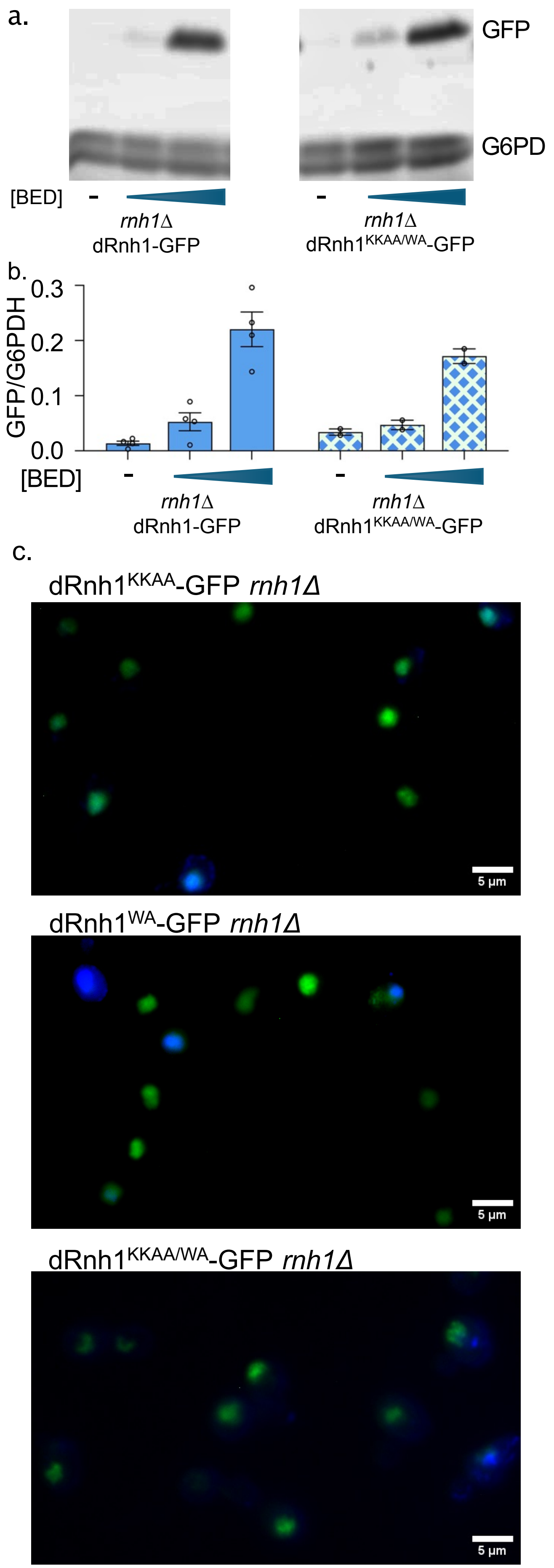

Fig. S5. (a)Representative Western blot of *rnh1Δ* strains expressing either dRnh1-GFP or dRnh1$^{KKAA/WA}$-GFP. Cells were induced for 4 hours in 0, 0.5, or 2nM BED. (b) Quantitation of Western blots depicted in a. Average pixel intensity of GFP band/average pixel intensity of G6PDH. (c) dRnh1^{KKAA}-GFP, dRnh1^{WA}-GFP, and dRnh1$^{KKAA/WA}$-GFP were all induced with 2nM BED for 4 hours. The dRnh1-GFP construct is indicated in green and the DAPI-labeled nucleus labeled in blue.
