# Supplementary material for: An improved cytological assay for R-loop detection in Saccharomyces cerevisiae utilizing a catalytically inactive RNase H
Source: G3 (Bethesda). 2025 Apr 10;15(6):jkaf072. doi: 10.1093/g3journal/jkaf072 (PMC12134985; doi:10.1093/g3journal/jkaf072)
Supplement: jkaf072_Supplementary_Data [file jkaf072_supplementary_data.zip › Figure_S6_G3-2024-405428.pdf]

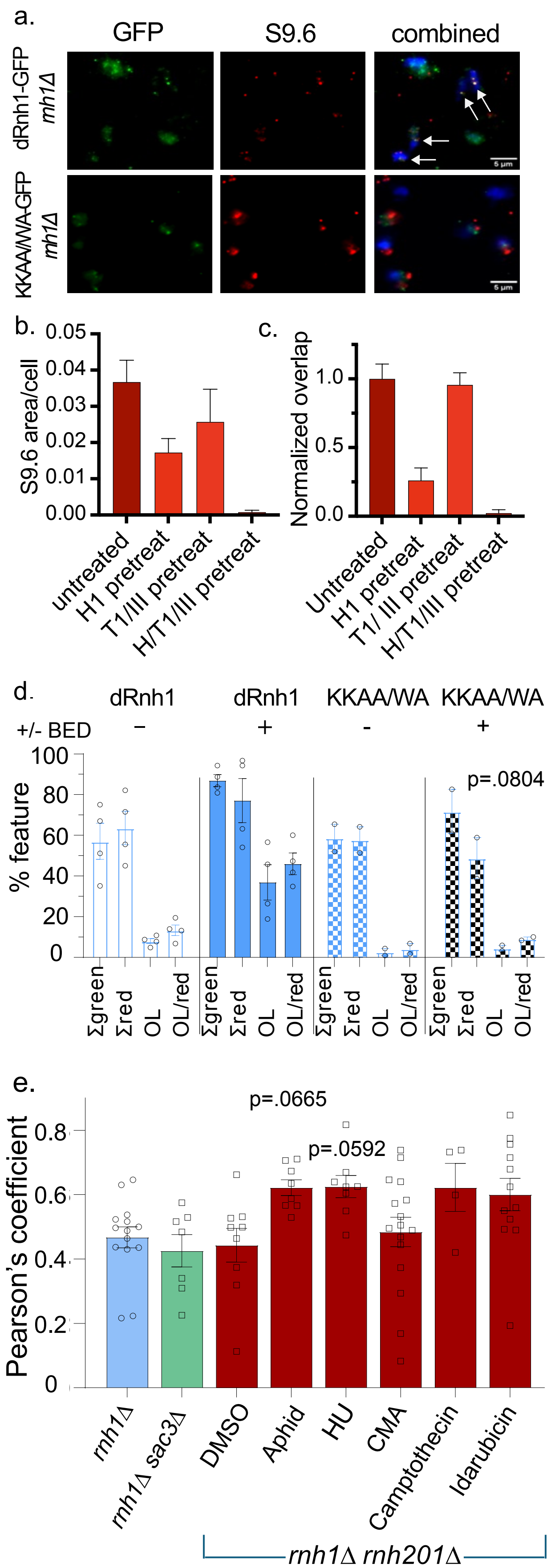

Fig. S6. dRnh1-GFP co-localizes with R-loops. (a) Representative images of chromatin spread IF. *rnh1Δ* cellsgrown for 4 hours in 2nM BED and harvested for S96 IF. dRnh1-GFP fluorescence indicated in green, R-loops as defined by S9.6 antibody in red, and DAPI-stained nucleus in blue. White arrows indicate examples of overlap between dRnh1-GFP and S9.6. (b) and (c) Effects of diagnostic nuclease pretreatment on S9.6 immunofluorescence. As described in Material and Methods, slides were either untreated prior to S9.6 addition, or treated and incubated with RNase HI (H1), a mixture of RNase T1 and RNase III (T1/III), or a mixture of all 3 RNases (H/T1/III). (b) quantifies the percentage of the field displaying S9.6 signal/#cells in said field. (c) quantifies the effect of nuclease pretreatment of S9.6/dRnh1-GFP overlap relative to the untreated sample.(d) Results of visual scoring of IF images. *rnh1Δ* strain was scored for the presence of dRnh1-GFP fluorescence (Σ green), S9.6 fluorescence (Σ red), and overlap between dRnh1-GFP/S9.6 signal (%OL). Results of this scoring are represented as a percentage of cells in the population displaying a given feature, as well as the percentage of cells displaying S9.6 signal that also display overlapping dRnh1-GFP signal (%OL/red). (e) Pearson's correlation coefficients of dRnh1-GFP/S9.6 overlap derived from chromatin spread IF images of *rnh1Δ*, *rnh1Δ sac3Δ*, and *rnh1Δ rnh201Δ* cells treated with 2nM BED for 4 hours. *rnh1Δ rnh201Δ* cells were additionally treated along with the BED induction with either 0.5% DMSO, 125µM aphidicolin, 25mM hydroxyurea, 250µM N-Methyl-β-carboline-3-carboxamide (CMA), 260µM camptothecin, or 10µM idarubicin.
