# Supplementary material for: An improved cytological assay for R-loop detection in Saccharomyces cerevisiae utilizing a catalytically inactive RNase H
Source: G3 (Bethesda). 2025 Apr 10;15(6):jkaf072. doi: 10.1093/g3journal/jkaf072 (PMC12134985; doi:10.1093/g3journal/jkaf072)
Supplement: jkaf072_Supplementary_Data [file jkaf072_supplementary_data.zip › Supplemental_Methods_G3-2024-405428.docx]

**Supplemental Methods**

**Chromatin Spread and S9.6 Immunofluorescence.** Strains were harvested for chromatin spreads and processed for immunofluorescence using a slightly modified protocol previously detailed by Koshland (Wahba, Amon et al. 2011) (http://mcb.berkeley.edu/labs/koshland/Protocols/MICROSCOPY/spreads.html). ~5 OD_600nm_ cells were harvested and washed in digest solution (80mM K_2_HPO_4_, 20mM KH_2_PO_4_, 1.2M sorbitol, 0.5mM MgCl_2_ pH~7.4), sonicated, and pelleted. Following resuspension in 0.32mL digest solution additionally containing 18.4mM DTT+ 123 ng/mL Zymolyase 20T, the cell walls were digested by incubation at 37^o^ C for 30 min. Digestion was stopped by addition of 1.5mL of ice-cold stop solution (100mM MES, 1mM EDTA, 0.5mM MgCl_2_ pH~ 6.4). Cells were centrifuged at 68 rcf for 8 minutes, resuspended in 50-200μL stop solution, and 20μL was spread on acid-washed slides. Cells were fixed by adding 40μL 4% paraformaldehyde in 3.4% sucrose, then immediately lysed with 80μL 1% Lipsol, and immediately fixed again with 80μL paraformaldehyde solution. Cells were spread across each slide with a micropipette tip and were allowed to dry overnight. Slides could be frozen at -20^o^ C or immediately used for immunofluorescence analysis. All strains were analyzed a minimum of two times.

To probe chromatin spreads for R-loops, slides were first rehydrated by immersion in PBS for 10 min at room temperature. To accommodate for RNase H pre-treatment control slides, 100μL of RNase H buffer (50mM Tris-Cl, 75mM KCl, 3mM MgCl2, 10mM DTT, pH ~8.3) was added to all slides, but only the RNase H pre-treatment slides received 2U/μL purified RNase H (NEB M0297S). All slides, regardless of RNase H pre-treatment, were incubated at 37°C for two hours in sealed humid chambers to prevent evaporation. Slides then were drained of excess liquid and washed with PBS. For select experiments (Fig. S6b, S6c), RNases T1 and III incubations were also included. Thermo RNase T1 (EN0541) and NEB ShortCut RNase III (M0245S) were diluted 1:300 and 1:4,000 respectively in reaction buffer (50mM Tris-Cl, 75mM KCl, 3mM MgCl2, 0.1% BSA, pH 7.6). Slides were similarly sealed in humid chambers and incubated at 37ºC for 2 hours. Following a final draining of excess liquid and wash in PBS, 200μL of blocking buffer (5% BSA+0.5% nonfat milk in PBS) was added onto slides for 10 min at room temperature. After draining excess blocking buffer, slides were treated overnight at room temperature with 100μL of blocking buffer containing 1:500 dilution of Kerafast mouse monoclonal α-RNA/DNA hybrid (S9.6) (Phillips, Garboczi et al. 2013). Slides were kept in sealed humid chambers during this step. The next morning, slides were immersed again in PBS for 10 min at RT and then treated with 100μL blocking buffer containing a 1:1,000 dilution of Invitrogen polyclonal goat α-mouse Alexa Fluor 555 (A32727) (Amini, Stojkov et al. 2018). Slides were incubated in the dark in humid chambers for 1 hr at RT and then immersed again in PBS for 10 min. Excess PBS was drained from slides and then slides were mounted with Invitrogen SlowFade Gold antifade reagent (S36936) with 1μg/mL DAPI. Coverslips were sealed onto the slides with clear nail polish and stored at -20^o^ C until use.

Fluorescence microscopy images of dRnh1-GFP and S9.6/R-loops were obtained using a Zeiss Axioskop 40 with Zeiss AxioCam HRM. To guarantee captured signal is within the upper detection limit of the equipment, the *rnh1Δ rnh201Δ* mutant induced with 2nM BED was used as a positive control to establish the optimal exposure time, image brightness, and image contrast setting on each channel. These settings were held constant for all other slides within the same experimental set.

**Chromatin Spread Immunofluorescence Analysis: Pearson’s Colocalization Coefficients.** Captured images in an experimental set were processed in parallel with FIJI image analysis software (Schindelin, Arganda-Carreras et al. 2012). Low-level background in all channels was subtracted using default settings (rolling ball radius 50 pixels). Pearson’s colocalization coefficient was derived from these modified images using the JACoP plugin (Bolte and Cordelieres 2006). Each coefficient was derived through analysis of an entire image (i.e. analysis of whole field with no ROI’s defined). To verify that the observed correlations were not an artifact of non-specific background fluorescence, a control Pearson’s value was calculated from images of *rnh1Δ rnh201Δ* +2nM BED spreads where red-channel images were vertically inverted relative to green-channel images (scrambled coefficient). Statistical change in Pearson’s coefficient between DMSO-treated *rnh1Δ rnh201Δ* cells and drug-treated cells was calculated using 1-way ANOVA using GraphPad PRISM software version 10.3.0 (Dunnett’s multiple comparison’s test, adjusted p-values reported).

**Chromatin Spread Immunofluorescence Analysis: Visual Analysis.** Chromatin spread images were captured and basic background subtracted as discussed above. Additional image thresholding was performed prior to visual scoring. Non-specific signal from secondary antibody was subtracted from all images by using no-primary control images to set threshold values. Autofluorescence in the green channel was similarly removed based on control cells grown in the absence of BED. Threshold values were held constant for all images within an experimental set. Channels were merged and each cell was visually inspected for presence of any visible dRnh1-GFP signal, S9.6 signal, and overlap between the two signals. Cells were deemed to have overlapping GFP/S9.6 signal only if the two signals overlap the same space *and* possess a similar size/pattern. Correlation between live-cell percentage of dRnh1-GFP positive-cells and dRnh1-GFP/S9.6 overlapping chromatin spreads determined with a simple linear regression analysis in GraphPad PRISM version 10.3.0.

**Chromatin Spread Immunofluorescence Analysis: Measuring TS9.6 Signal Area** Total S9.6 immunofluorescence signal per cell was analyzed in ImageJ/FIJI. Chromatin spread images were thresholded as discussed in the previous section. Area of S9.6 fluorescence above threshold across an entire image was recorded using the ImageJ measure function. S9.6 area was then divided by number of DAPI-staining nuclei observed in the image.
