# Supplementary material for: An improved cytological assay for R-loop detection in Saccharomyces cerevisiae utilizing a catalytically inactive RNase H
Source: G3 (Bethesda). 2025 Apr 10;15(6):jkaf072. doi: 10.1093/g3journal/jkaf072 (PMC12134985; doi:10.1093/g3journal/jkaf072)
Supplement: jkaf072_Supplementary_Data [file jkaf072_supplementary_data.zip › Table_S1_G3-2024-405428.pdf]

Table S1: Strain List

Strain #	Genotype	Source
UPY1325	MATα *leu2-3,112 ura3-52 his3Δ200 trp1Δ901 suc2Δ9 lys2-801* GAL *snq2*::KanMX *pdr5-Δ1*::hisG *yor1-1*::hisG	S. Moye-Rowley (Kolaczkowski, Kolaczowska et al. 1998)
UPY1804	MATα *leu2-3,112 his3Δ200 trp1Δ901 suc2Δ9 lys2-801* GAL *snq2*::HygR pdr5Δ1::hisG *yor1-1*::hisG act1_GEV::NatR *rnh1Δ*::KANMX *ura3-52*::IVY-*rnh1D264N* URA3 (pUP1447)	This study
UPY1831	MATα *leu2-3,112 ura3-52* pGal-Ivy-*RNH1*::*URA3* (pUP1446) *his3Δ200 trp1Δ901 suc2Δ9 lys2-801* GAL *snq2*::KanMX *pdr5Δ1*::hisG *yor1-1*::hisG act1_GEV::NatR *MED7*-Scarlet::HygR	This study
UPY1843	MATα *leu2-3,112 ura3-52* pGal-*rnh1D264N*-EGFP::*URA3* (pUP1456.1) *his3Δ200 trp1Δ901 suc2Δ9 lys2-801* GAL *snq2*::HygR *pdr5Δ1*::hisG *yor1-1*::hisG act1_GEV::NatR	This study
UPY1859	MATα *leu2-3,112 ura3-52* pGal-*rnh1D264N*-EGFP::*URA3* (pUP1456.1) *his3Δ200 trp1Δ901 suc2Δ9 lys2-801* GAL *snq2*::HygR *pdr5Δ1*::hisG *yor1-1*::hisG act1_GEV::NatR *rnh1Δ*::His6MX	This study
UPY1865	MATα *leu2-3,112 ura3-52* pGal-*RNH1*-EGFP::*URA3* (pUP1453.1) *his3Δ200 trp1Δ901 suc2Δ9 lys2-801* GAL *snq2*::HygR *pdr5Δ1*::hisG *yor1-1*::hisG act1_GEV::NatR *rnh201Δ*::KANMX *rnh1Δ*::His6MX	This study
UPY1867	MATα *leu2-3,112 ura3-52* pGal-*rnh1D264N*-EGFP::*URA3* (pUP1456.1) *his3Δ200 trp1Δ901 suc2Δ9 lys2-801* GAL *snq2*::HygR *pdr5Δ1*::hisG *yor1-1*::hisG act1_GEV::NatR *rnh201Δ*::KANMX *rnh1Δ*::His6MX	This study
UPY1874	MATα *leu2-3,112 ura3-52* pGal-*rnh1D264N*-EGFP::*URA3* (pUP1456.1) *his3Δ200 trp1Δ901 suc2Δ9 lys2-801* GAL *snq2*::HygR *pdr5Δ1*::hisG *yor1-1*::hisG act1_GEV::NatR *rnh1Δ*::His6MX *btn2Δ*::KanMX	This study
UPY1876	MATα *leu2-3,112 ura3-52* pGal-*rnh1D264N*-EGFP::*URA3* (pUP1456.1) *his3Δ200 trp1Δ901 suc2Δ9 lys2-801* GAL *snq2*::HygR *pdr5Δ1*::hisG *yor1-1*::hisG act1_GEV::NatR *rnh1Δ*::His6MX VHL-mCherry::*LEU2* (pUP1460)	This study
UPY1878	MATα *leu2-3,112 ura3-52* pGal-*rnh1D264N*-EGFP::*URA3* (pUP1456.1) *his3Δ200 trp1Δ901 suc2Δ9 lys2-801* GAL *snq2*::HygR *pdr5Δ1*::hisG *yor1-1*::hisG act1_GEV::NatR *rnh1Δ*::His6MX sac3Δ::KanMX	This study
UPY1883	MATα *leu2-3,112 ura3-52* *rnh1^{KKAA}D264N*-EGFP::*URA3* (pUP1462.1) *his3Δ200 trp1Δ901 suc2Δ9 lys2-801* GAL *snq2*::HygR *pdr5Δ1*::hisG *yor1-1*::hisG act1_GEV::NatR *rnh1Δ*::His6MX	This study

UPY1884	MATα *leu2-3,112 ura3-52 rnh1*^{WA}*D264N*-EGFP::*URA3* (pUP1463.1) *his3Δ200 trp1Δ901 suc2Δ9 lys2-801* GAL *snq2*::HygR *pdr5Δ1*::hisG *yor1-1*::hisG act1_GEV::NatR *rnh1Δ*::His6MX	This study
UPY1885	MATα *leu2-3,112 ura3-52 rnh1*$^{KKAA/WA}$*D264N*-EGFP::*URA3* (pUP1464.1) *his3Δ200 trp1Δ901 suc2Δ9 lys2-801* GAL *snq2*::HygR *pdr5Δ1*::hisG *yor1-1*::hisG act1_GEV::NatR *rnh1Δ*::His6MX	This study
UPY1890	MATα *leu2-3,112 ura3-52* pGal-IVY-*rnh1D264N*::*URA3* (pUP1447.1) *his3-Δ200 trp1Δ901 suc2Δ9 lys2-801* GAL *snq2*::KanMX *pdr5Δ1*::hisG *yor1-1*::hisG act1_GEV::NatR VHL-mCherry::*LEU2* (pUP1460) *rnh1Δ*::His6MX	This study
UPY1927	MATα *leu2-3,112 ura3-52* pGal-*rnh1D264N*-EGFP::*URA3* (pUP1456.1) *his3-Δ200 trp1Δ901 suc2Δ9 lys2-801* GAL *snq2*::KanMX *pdr5Δ1*::hisG *yor1-1*::hisG act1_GEV::NatR VHL-mCherry::*LEU2* (pUP1460) *rnh1Δ*::His6MX	This study
