# Supplementary material for: An improved cytological assay for R-loop detection in Saccharomyces cerevisiae utilizing a catalytically inactive RNase H
Source: G3 (Bethesda). 2025 Apr 10;15(6):jkaf072. doi: 10.1093/g3journal/jkaf072 (PMC12134985; doi:10.1093/g3journal/jkaf072)
Supplement: jkaf072_Supplementary_Data [file jkaf072_supplementary_data.zip › Table_S2_G3-2024-405428.pdf]

Table S2: Plasmid List

Plasmid #	Gene	Plasmid type	Bact. marker	Yeast marker	Source
pUP1135	ccdb	Donor plasmid	KanR	G418	(Alberti, Gitler et al. 2007)
pUP1230	pGal-Rnh1	Integrating	AmpR	URA	This study
pUP1305	Act1-GEV	Integrating	AmpR	NatR	(McIsaac, Silverman et al. 2011)
pUP1306	Ivy GFP	Integrating	AmpR	His	(Slubowski, Funk et al. 2015)
pUP1417	*RNH1*	Entry vector	KanR	G418	This study
pUP1440	*dRNH1*	Entry vector	KanR	G418	This study
pUP1443	pGal-EGFP-ccbd	Destination vector	AmpR	URA	(Alberti, Gitler et al. 2007)
pUP1445	pGal-*dRNH1*	Integrating	AmpR	URA	This study
pUP1447	pGal-Ivy-*dRNH1*	Integrating	AmpR	URA	This study
pUP1453	pGal-*RNH1*-EGFP	Integrating	AmpR	URA	This study
pUP1455	pGal-*RNH1*-EYFP	Integrating	AmpR	URA	This study
pUP1456	pGal-*dRNH1*-EYFP	Integrating	AmpR	URA	This study
pUP1460	pGal-mCherry-VHL	2μ	AmpR	LEU	(Mathew, Tam et al. 2017)
pUP1462	pGal-$dRNH1^{KKAA}$-EYFP	Integrating	AmpR	URA	This study
pUP1463	pGal-$dRNH1^{WA}$-EYFP	Integrating	AmpR	URA	This study
pUP1464	pGal-$dRNH1^{KKAA/WA}$-EYFP	Integrating	AmpR	URA	This study
