# Supplementary material for: An improved cytological assay for R-loop detection in Saccharomyces cerevisiae utilizing a catalytically inactive RNase H
Source: G3 (Bethesda). 2025 Apr 10;15(6):jkaf072. doi: 10.1093/g3journal/jkaf072 (PMC12134985; doi:10.1093/g3journal/jkaf072)
Supplement: jkaf072_Supplementary_Data [file jkaf072_supplementary_data.zip › Table_S3_G3-2024-405428.pdf]

Table S3: Oligo List

#	Sequence	Associated Gene/Plasmid
1486	TTCTGGTGTCTTGATTGCCGA	*sac3Δ*
1487	AGCAGTTTATAACTTTTTGGCCTCT	*sac3Δ*
1899	AATCAAAACTaATTCAGAGTACGTAAC	pUP1440
1900	TGATAATTTACTTTTTCCTTTTCATTAG	pUP1440
1917	GGTGGACCAGGTGGTGGACA	pUP1447
1918	TTTGTCCCGGGGGATCCACTAG	pUP1447
1919	ttctagaactagtggatcccccgggacaaaATGGTTTCTAAAGGTGAAGAGCTATTCAC	pUP1447
1920	acttgtttgatgtccaccacctggtccaccCGCGGCTGTGACAAATTCGAG	pUP1447
1931	CATGTATAAAGCACACTGCACCTAC	*rnh1Δ*
1932	GATGAAAAACTCGAAGAAATTGAAA	*rnh1Δ*
1933	AATTGACAGGAAACAAAACTGAGAC	*rnh201Δ*
1934	ACAAAGGCAGACATAGTACGCTAAT	*rnh201Δ*
1935	TGCAATTTATgcagcaTTTAACAGCTACGAGC	pUP1462
1936	CCACCATAACCATCAACTTG	pUP1462
1937	CTATAATACAgctAATGAATGTAAAAACCAAGTTGATGGTTATGG	pUP1463
1938	ATCCCAGTTTCCCTGCCC	pUP1463
1945	CGAGAGTTGTATCCAGTTTTCTTGT	*btn2Δ*
1946	CTTGCCATTCCACCATAAAATACT	*btn2Δ*
