# Supplementary material for: An improved cytological assay for R-loop detection in Saccharomyces cerevisiae utilizing a catalytically inactive RNase H
Source: G3 (Bethesda). 2025 Apr 10;15(6):jkaf072. doi: 10.1093/g3journal/jkaf072 (PMC12134985; doi:10.1093/g3journal/jkaf072)
Supplement: jkaf072_Supplementary_Data [file jkaf072_supplementary_data.zip › Table_S4_G3-2024-405428.pdf]

Table S4: Compound List

Compound name	Conc. in drug screen	Supplier	Product/Catalog #
Mouse α-GFP Ab	N/A	Roche	11814460001
α-Mouse IgG HRP-linked Ab	N/A	Sigma	NA931V
Mouse α-RNA/DNA hybrid (S9.6)	N/A	Kerafast	ENH001
α-Mouse Alexa Fluor™ Plus 555	N/A	Invitrogen	A32727
Rabbit α-G6PDH Ab	N/A	Sigma	A9521
α-Rabbit IgG HRP-linked Ab	N/A	Sigma	NA934V
Aphidicolin	125 µM	Sigma	A0781
β-carboline-3-carboxylic acid N-methylamide (CMA)	250 µM	Sigma	E006
β-estradiol	0, 0.5, 1, 2nM	Sigma	E8875
Camptothecin	260 µM	Sigma	C9911
4′,6-diamidino-2-phenylindole (DAPI)	N/A	Sigma	10236276001
Idarubicin	10 µM	Sigma	I1656
Ellipticine	100 µM	EMD Millipore	324688
Hydroxyurea	25, 50, 200 mM	Sigma	H8627
Methyl methanesulfonate	0.03%	Sigma	129925
Nourseothricin	N/A	Jena Bioscience	AB-102XL
ProSignal® Dura Low-Femtogram ECL Reagent	N/A	Genesee Scientific	20-301
RNase H	N/A	NEB	M0297S
RNase T1	N/A	Thermo	EN0541
ShortCut RNase III	N/A	NEB	M0245S
SlowFade Gold antifade	N/A	ThermoFisher	S36936
SuperSignal™ West Pico PLUS	N/A	ThermoFisher	34577
